# Coumarin Reduces Aluminum-Induced Inhibition of Growth and Photosynthesis in *Citrus grandis* by Reducing Tissue Al Concentration and Maintaining Nutrient and Redox Homeostasis

**DOI:** 10.3390/plants15111694

**Published:** 2026-05-30

**Authors:** Hui Yang, Rong-Rong Xie, Tian-Tian Xia, Liang-Yuan Tong, Ti Wu, Xin Ye, Zeng-Rong Huang, Lin-Tong Yang, Li-Song Chen

**Affiliations:** 1 College of Resources and Environment, Fujian Agriculture and Forestry University, Fuzhou 350002, China; 52308031045@fafu.edu.cn (H.Y.); rongrxie@163.com (R.-R.X.); 15982655984@163.com (T.-T.X.); tongliangyuan2002@163.com (L.-Y.T.); huangzengrong@fafu.edu.cn (Z.-R.H.); talstoy@fafu.edu.cn (L.-T.Y.); 2 College of Environmental and Biological Engineering, Putian University, Putian 351100, China

**Keywords:** aluminum toxicity, chlorophyll *a* fluorescence transient, coumarin, DPPH, ROS, soluble sugars, Sour pummelo

## Abstract

No data to date are available on the underlying mechanisms by which coumarin (COU) alleviates plant aluminum (Al) toxicity. *Citrus grandis* (L.) Osbeck seedlings were submitted to 0 (Al0) or 1.2 (Al1.2 or Al^3+^ toxicity) mM AlCl_3_·6H_2_O and 0 (COU0) or 50 (COU50) μM COU for 18 weeks. The results demonstrated that COU50 attenuated Al1.2-induced decreases of seedling growth, chlorophyll (Chl) level, and CO_2_ assimilation (A_CO2_) and impairment of the photosynthetic electron transport chain (PETC). Further analysis suggested that reduced tissue Al concentration and enhanced capability to maintain nutrient and redox homeostasis played a role in COU-mediated amelioration of seedling growth inhibition, leaf Chl and A_CO2_ decline, and PETC impairment. Notably, seedlings treated with COU0 showed some adaptive responses to Al1.2. For example, Al1.2 decreased the biosynthesis and accumulation of proteins and amino acids to meet the increased need for energy; increased the diphenylpicrylhydrazyl (DPPH) scavenging activity and phenolic compound accumulation to meet the elevated demand for reactive oxygen species (ROS) and Al detoxification; and increased the accumulation of soluble sugars (glucose, fructose, and sucrose) to meet the augmented demand for ROS scavenging and energy. To conclude, the research revealed some mechanisms for COU-mediated amelioration of plant Al^3+^ toxicity.

## 1. Introduction

In acidic soil with a pH below 5.0, aluminum (Al) primarily exists as Al^3+^ and is detrimental to most crops [1]. Al^3+^ toxicity first arrests root elongation, subsequently impairs water and nutrient absorption, and ultimately reduces crop growth and yield [2,3]. Al^3+^ toxicity-induced decline in leaf CO_2_ assimilation (A_CO2_) is a common phenomenon in many higher plants, including *Citrus* spp. [4,5]. Al^3+^ toxicity can induce over-accumulation of reactive oxygen species (ROS) in many plants, including *Citrus* [6]. Over-accumulation of ROS will cause oxidative damage, thereby affecting key biomolecules including nucleic acids, proteins, carbohydrates, and lipids [2,7]. Al^3+^ toxicity is the main factor affecting crop growth and yield in acidic soil, occupying ~30% of the world’s ice-free land area [8] and ~23% of China’s total land area [9]. Unfortunately, the acidification of the world’s arable land is showing an exponential growth trend [10]. Al^3+^ toxicity is considered one of the main factors affecting crop productivity and the second largest abiotic stress after drought that affects crop productivity worldwide [11]. Therefore, revealing the mechanisms underlying Al^3+^ tolerance and developing new approaches to mitigate Al^3+^ toxicity are of both theoretical and agronomic significance for improving crop productivity in acidic soil.

Exogenous application of compounds enhances a crop’s tolerance to metal toxicity, opening new pathways to reduce the toxicity of Al and heavy metals (HMs) to crops [12,13,14,15,16]. Stress conditions typically lead to the accumulation of secondary metabolites (SMs), including phenolic compounds, alkaloids, and terpenes, which can protect plants against abiotic (metal, salinity, and drought) and biotic stresses [17,18,19,20]. As a class of plant-derived phenolic compounds produced via the phenylpropanoid pathway, coumarins (COUs) have been considered as iron (Fe)-mobilizing compounds secreted by plant roots, helping to absorb Fe from Fe-limited soils [21]. It was reported that excessive HMs (manganese (Mn), copper (Cu), and zinc (Zn)) led to Fe deficiency chlorosis of younger leaves [15,16,22]. Supplementation of fraxetin (also known as 7,8-dihydroxy-6-methoxycoumarin; a natural derivative of COUs) complemented the Fe-deficient phenotypes of the *f6′h1-1* (*F6′H* encodes a feruloyl-CoA 6-hydroxylase) and *s8h* (*S8H* encodes a scopoletin 8-hydroxylase) *Arabidopsis thaliana* (L.) Heynh. mutants [23]. Applying 50 μM coumarin (COU, also known as 1,2-benzopyrone, a plant-derived compound with significant ROS scavenging ability) to trifoliate orange (*Poncirus trifoliata* Raf.) roots mitigated Fe deficiency yellowing of younger leaves caused by high pH [24,25]. In addition to their function in Fe acquisition [26,27], COUs have been shown to play a role in protecting plants from (a)biotic stresses, including metal stress [15,16,28]. Beesley et al. [28] observed that engineered accumulation of COUs (scopoletin and its glucoside scopolin and esculin) in transgenic soybean (*Glycine max* L.) plants overexpressing an *A. thaliana feruloyl-CoA 6-hydroxylase 1* (*AtF6′H1*) attenuated mycotoxin-induced oxidative damage and cell death. Saleh and Madany [29] indicated that the mitigation of COU to NaCl-induced growth inhibition of wheat (*Triticum aestivum* L.) seedlings involved enhanced antioxidant defense system and osmoregulation process. A recent work from our laboratory showed that application of COU to ‘Xuegan’ (*Citrus sinensis* (L.) Osbeck) roots attenuated Cu excess-triggered decline in seedling growth, A_CO2_, and chlorophyll (Chl) concentration, as well as impairment to the photosynthetic electron transport chain (PETC) via lessening Cu absorption and oxidative stress and keeping the homeostasis of other nutrients [15]. A study with sesame (*Sesamum indicum* L.) seedlings indicated that exogenous application of COU attenuated Mn excess-induced decreases in seedling growth, A_CO2_, and Chl level via reducing tissue Mn concentration, maintaining other nutrient homeostasis, and preventing oxidative stress [16]. Ashraf et al. [13] reported that foliar application of COU in castor bean (*Ricinus communis* L.) seedlings mitigated Cu-, chromium (Cr)-, and nickel (Ni)-induced decreases in growth, foliar concentration of Chl, and root and leaf concentrations of potassium (K), calcium (Ca), nitrogen (N), and phosphorus (P); increases in root and leaf concentrations of Cu, Cr, and Ni; and oxidative stress due to reduced ROS production and accumulation and augmented activities of antioxidant enzymes, concentrations of total phenolics (TPs) and total flavonoids, and diphenylpicrylhydrazyl (DPPH)-scavenging activity. Notably, COU increased the foliar concentrations of total soluble proteins (TSPs), total free amino acids (TFAAs), non-reducing sugar, reducing sugar, and total soluble sugar (TSS) under Cu, Cr, and Ni stress. This drives us to hypothesize that exogenous application of COU mitigated Al^3+^ toxicity-induced decreases in growth, A_CO2_, and Chl level via reducing tissue Al concentration, maintaining other nutrient (concentrations, distribution, and balance) homeostasis, and preventing oxidative stress. To the best of our knowledge, no data to date are available on COU-mediated regulation of growth, A_CO2_, Chl, ROS production and scavenging, and Al and nutrient absorption in plants under Al^3+^ toxicity.

Most *Citrus* trees are cultivated on acidic soil containing high active Al^3+^ [30,31]. Reducing Al^3+^ availability has been suggested to be the critical nutrient management strategy for attenuating soil acidification and enhancing *Citrus* root growth in acidic soil [32]. Unfortunately, soil acidification often occurs in China’s *Citrus* orchards [30,31,33]. It was reported that in the main *Citrus*-producing areas of China, more than 49% of *Citrus* orchards had a soil pH below 4.8 [30]. Here, we examined the roles of COU in alleviating the toxicity of Al^3+^ on ‘Sour pummelo’ (*Citrus grandis* (L.) Osbeck) seedlings: insight from Al and nutrient uptake; growth and biomass distribution; leaf photosynthetic performance (gas exchange, Chl *a* fluorescence (OJIP) transient, and Chl); and nonstructural carbohydrates (NCs), ROS metabolism (ROS production, malondialdehyde (MDA), antioxidant enzymes, and DPPH scavenging activity), and related physiological parameters in leaves and roots. The objective of the research was to validate the above hypothesis.

## 2. Results

### 2.1. Seedling Growth Characteristics

**Figure 1:**
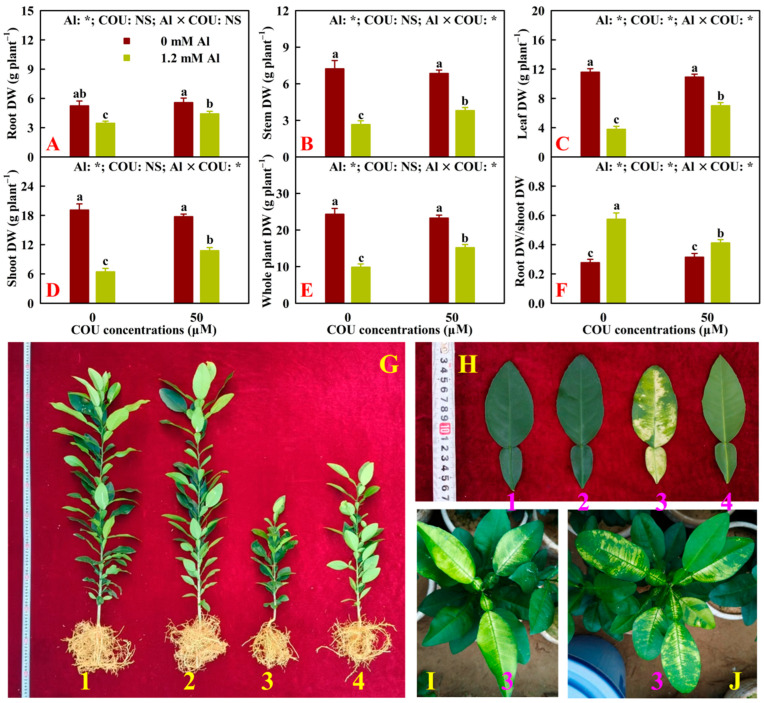
Effects of Al–COU treatments on the mean (±SE, *n* = 10) root dry weight (DW, (**A**)), stem DW (**B**), leaf DW (**C**), shoot DW (**D**), whole plant DW (**E**), and root DW/shoot DW ratio (R/S, (**F**)), as well as growth (**G**–**J**) of ‘Sour pummelo’ seedlings. Bars with different letters are significantly different at *p* ≤ 0.05. Al: *, COU: *, and Al × COU: * represent that the *F* values for Al, COU, and Al × COU are significant at *p* ≤ 0.05. COU: NS and Al × COU: NS represent that the *F* values for COU and Al × COU are not significant (*p* > 0.05). 1, Al0 + COU0; 2, Al0 + COU50; 3, Al1.2 + COU0; 4, Al1.2 + COU50.

It was found that Al^3+^ toxicity (Al1.2) significantly lowered root dry weight (DW) by 34.2% and 20.8%, stem DW by 63.2% and 44.6%, leaf DW by 67.4% and 35.9%, shoot DW by 66.3% and 39.2%, and whole plant DW by 59.4% and 34.8% at COU0 and COU50, respectively (Figure 1A–E). Because root DW was reduced to a lesser degree than shoot DW at Al1.2, root DW/shoot DW (R/S) increased in response to Al1.2, with a greater increment at COU0 than at COU50 (Figure 1F).

The research demonstrated that COU50 attenuated the mottled bleach leaves that occurred in Al1.2-treated seedlings (Figure 1G–J). Mottled bleach leaves have been observed in leaves of low pH-exposed ‘Xuegan’ and ‘Sour pummelo’ seedlings [34].

### 2.2. Leaf Photosynthetic Performances

**Figure 2:**
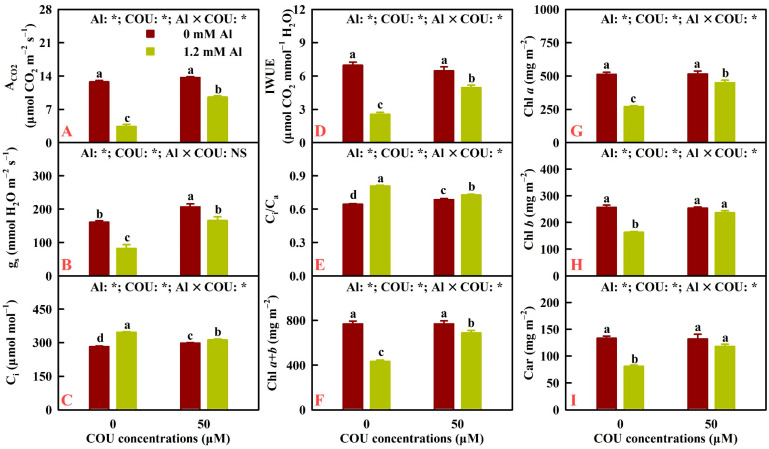
Impacts of Al–COU treatments on the mean (±SE, *n* = 4) A_CO2_ (**A**), g_s_ (**B**), C_i_ (**C**), IWUE (**D**), C_i_/C_a_ (**E**), Chl *a+b* (**F**), Chl *a* (**G**), Chl *b* (**H**), and Car (**I**) in leaves. Bars with different letters are significantly different at *p* ≤ 0.05. Al: *, COU: *, and Al × COU: * represent that the *F* values for Al, COU, and Al × COU are significant at *p* ≤ 0.05. Al × COU: NS represents that the *F* value for Al × COU is not significant (*p* > 0.05). Chl, chlorophyll; Car, carotenoids; A_CO2_, CO_2_ assimilation; C_i_, intercellular CO_2_ concentration; C_i_/C_a_, ratio of intercellular to ambient CO_2_ concentration; g_s_, stomatal conductance; IWUE, instantaneous water use efficiency.

Al^3+^ toxicity significantly reduced A_CO2_ by 73.6% and 30.0%, instantaneous water use efficiency (IWUE) by 63.4% and 23.1%, and stomatal conductance (g_s_) by 49.0% and 19.6% at COU0 and COU50, respectively; however, it significantly increased intercellular CO_2_ concentration (C_i_) by 22.9% and 5.1% and ratio of intercellular to ambient CO_2_ concentration (C_i_/C_a_) by 25.4% and 6.2% at COU0 and COU50, respectively (*p* ≤ 0.05) (Figure 2A–E).

It was found that Al1.2 significantly lessened Chl *a+b* level by 43.5% and 10.6%, and Chl *a* level by 47.1% and 12.5% at COU0 and COU50, respectively, as well as Chl *b* level by 36.5% and Car level by 39.0% at COU0; however, it did not significantly change Chl *b* and Car levels at COU50 (Figure 2F–I).

**Figure 3:**
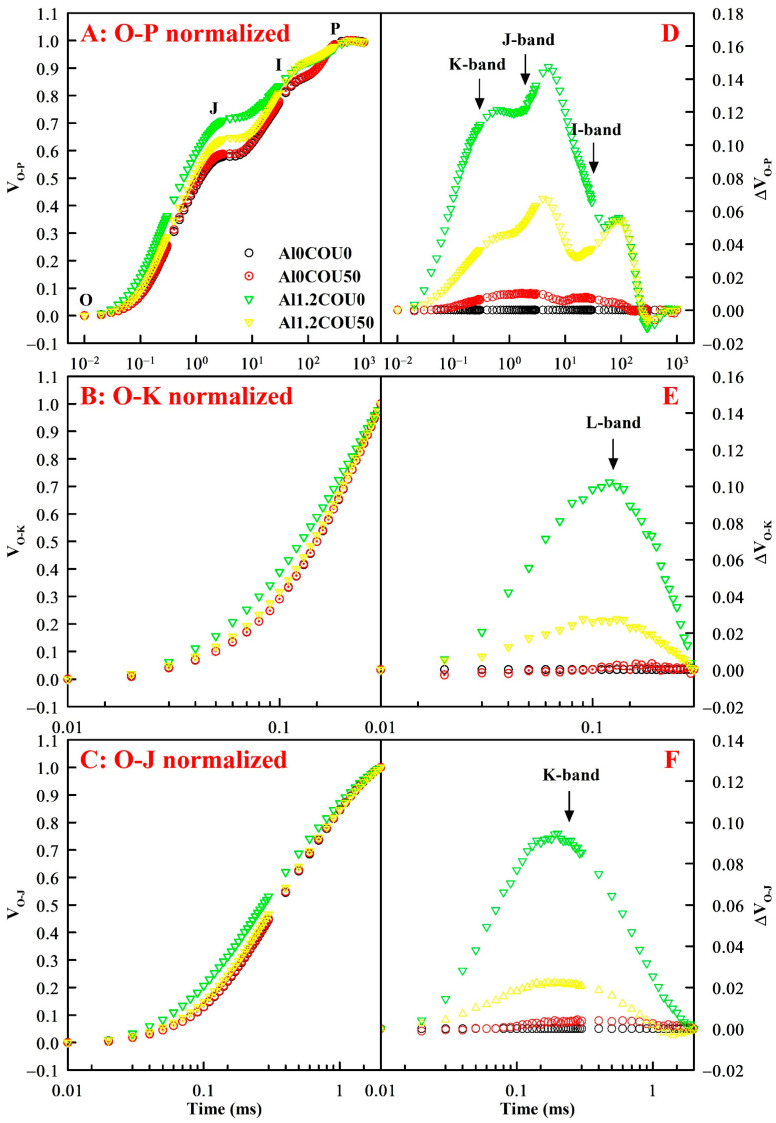
Effects of Al–COU treatments on the mean (*n* = 10) OJIP transients expressed as the kinetics of relative variable fluorescence: between F_o_ and F_m_: V_O-P_ = (F_t_ − F_o_)/(F_m_ − F_o_) (**A**) and the differences of the four samples to the reference sample treated with Al0COU0 (ΔV_O-P_, (**D**)); between F_o_ and F_300μs_: V_O-K_ = (F_t_ − F_o_)/(F_300μs_ − F_o_) (**B**) and the differences of the four samples to the reference sample (ΔV_O-K_, (**E**)); and between F_o_ and F_J_: V_O-J_ = (F_t_ − F_o_)/(F_J_ − F_o_) (**C**) and the differences of the four samples to the reference sample (ΔV_O-J_, (**F**)). F_t_, fluorescence at time *t* after onset of actinic illumination; F_o_, minimum fluorescence; F_m_, maximum fluorescence; F_J_, fluorescence intensity at the J-step (2 ms); F_300μs_, fluorescence intensity at 300 μs.

The research showed that the OJIP transients in leaves of Al1.2-treated seedlings (LAl1.2) had a positive ΔL-band at ~130 μs, ΔK-band at 300 μs, ΔI-band at 30 ms, and ΔJ-band at 2 ms relative to the OJIP transients in leaves of Al0COU0-treated seedlings (LAl0COU0), and these positive bands were alleviated by the addition of COU (Figure 3).

**Figure 4:**
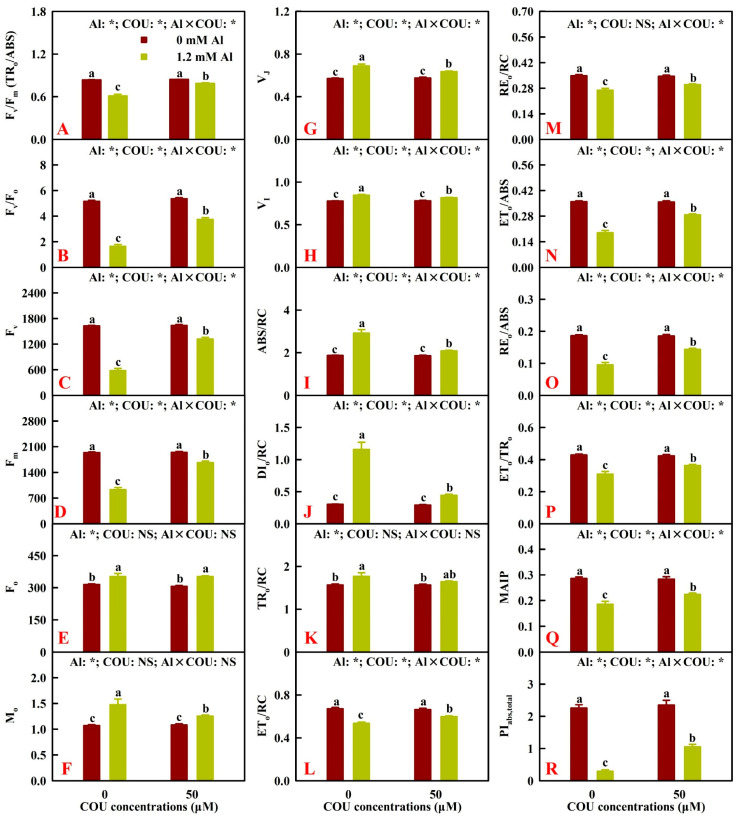
Effects of Al–COU treatments on the mean values (±SE, *n* = 10) of F_v_/F_m_ (**A**), F_v_/F_o_ (**B**), F_v_ (**C**), F_m_ (**D**), F_o_ (**E**), M_o_ (**F**), V_J_ (**G**), V_I_ (**H**), ABS/RC (**I**), DI_o_/RC (**J**), TR_o_/RC (**K**), ET_o_/RC (**L**), RE_o_/RC (**M**), ET_o_/ABS (**N**), RE_o_/ABS (**O**), ET_o_/TR_o_ (**P**), MAIP (**Q**), and PI_abs,total_ (**R**) in leaves. Bars with different letters are statistically significant (*p* ≤ 0.05). Al: *, COU: *, and Al × COU: * represent that the *F* values for Al, COU, and Al × COU are significant at *p* ≤ 0.05. COU: NS and Al × COU: NS represent that the *F* values for COU and Al × COU are not significant (*p* > 0.05). TR_o_/ABS or F_v_/F_m_, maximum quantum yield of primary photochemistry; F_v_/F_o_, maximum primary yield of photochemistry of photosystem II; F_v_, maximum variable fluorescence; F_m_, maximum fluorescence; F_o_, minimum fluorescence; M_o_, approximated initial slope (in ms^−1^) of the fluorescence transient *V* = *f*(*t*); V_J_, relative variable fluorescence at the J-step (2 ms); V_I_, relative variable fluorescence at the I-step (30 ms); ABS/RC, absorption flux per reaction center (RC) at *t* = 0; DI_o_/RC, dissipated energy flux per RC at *t* = 0; TR_o_/RC, trapped energy flux per RC at *t* = 0; ET_o_/RC, electron transport flux per RC at *t* = 0; RE_o_/RC, reduction of end acceptors at PSI electron acceptor side per RC at *t* = 0; ET_o_/ABS, quantum yield for electron transport at *t* = 0; RE_o_/ABS, quantum yield for the reduction of end acceptors of photosystem I per photon absorbed at *t* = 0; ET_o_/TR_o_, probability (at time 0) that a trapped exciton moves an electron into the electron transport chain beyond Q_A_^−^; MAIP, maximum amplitude of IP phase; PI_abs,total_, total performance index.

The research showed that COU50 attenuated Al1.2-induced reductions in F_v_/F_o_, F_v_/F_m_, F_m_, F_v_, ET_o_/RC_o_, ET_o_/TR_o_, RE_o_/ABS, RE_o_/RC, ET_o_/ABS, PI_abs,total_, and MAIP, and increments in F_o_, M_o_, ABS/RC, DI_o_/RC, V_J_, V_I_, and TR_o_/RC. Notably, R_o_/RC at COU50 was not significantly altered by Al1.2 (Figure 4).

### 2.3. Aluminum and Nutrient Profiles

**Figure 5:**
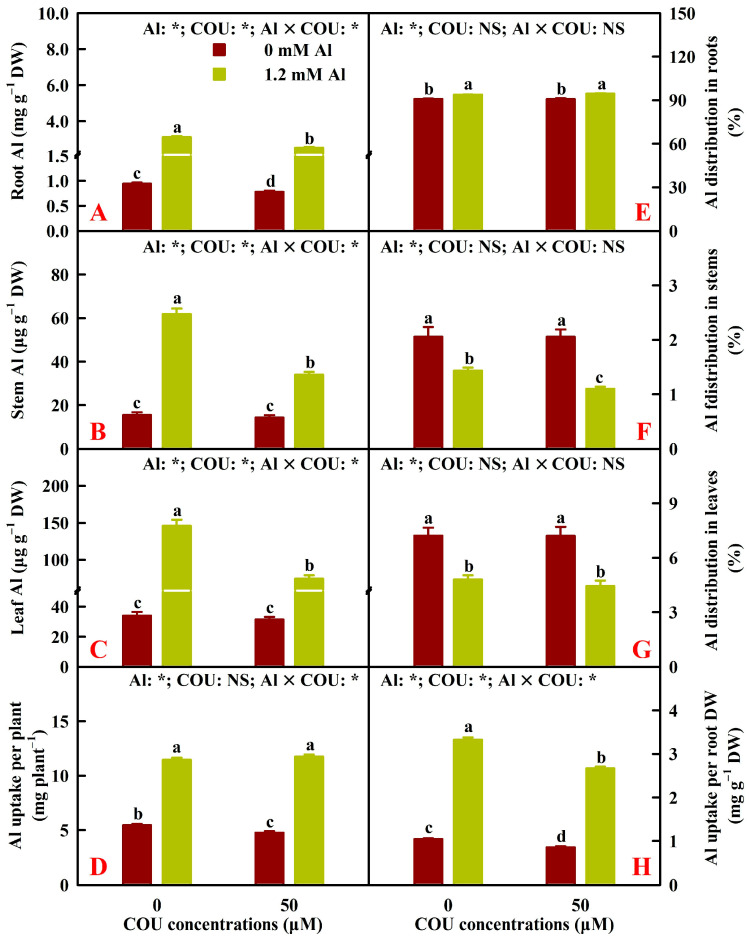
Impacts of Al–COU treatments on the mean (±SE, *n* = 4) Al concentrations in roots (**A**), stems (**B**), and leaves (**C**); Al UPP (**D**); Al distributions in roots (**E**), stems (**F**), and leaves (**G**); and Al UPR (**H**) of ‘Sour pummelo’ seedlings. Bars with different letters are significantly different at *p* ≤ 0.05. Al: *, COU: *, and Al × COU: * represent that the *F* values for Al, COU, and Al × COU are significant at *p* ≤ 0.05. COU: NS and Al × COU: NS represent that the *F* values for COU and Al × COU are not significant (*p* > 0.05). UPP, uptake per plant; UPR, uptake per root DW.

Figure 5 shows the responses of the Al profile to Al–COU treatments. The results showed that Al1.2 increased root Al level by 229.1% and 223.9%, leaf Al level by 327.7% and 137.6%, and stem Al level by 297.5% and 136.7%, and Al UPR by 218.4% and 211.2% at COU0 and COU50, respectively; and that root, leaf, and stem Al concentrations and Al UPR at Al1.2 were higher at COU0 than at COU50. Additionally, Al1.2 significantly increased Al UPP by 109.6% and 146.3%, and Al distribution in roots by 3.3% and 4.1% at COU0 and COU50, respectively; meanwhile, both Al UPP and Al distribution in roots at Al1.2 did not significantly differ between COU0 and COU50. By contrast, Al1.2 significantly decreased Al distribution in stems by 30.3% and 46.7% and Al distribution in leaves by 33.4% and 38.3% at COU0 and COU50, respectively. COU50 did not significantly affect Al distribution in leaves at Al1.2, but Al distribution in stems at Al1.2 was significantly lower at COU50 than at COU0.

**Figure 6:**
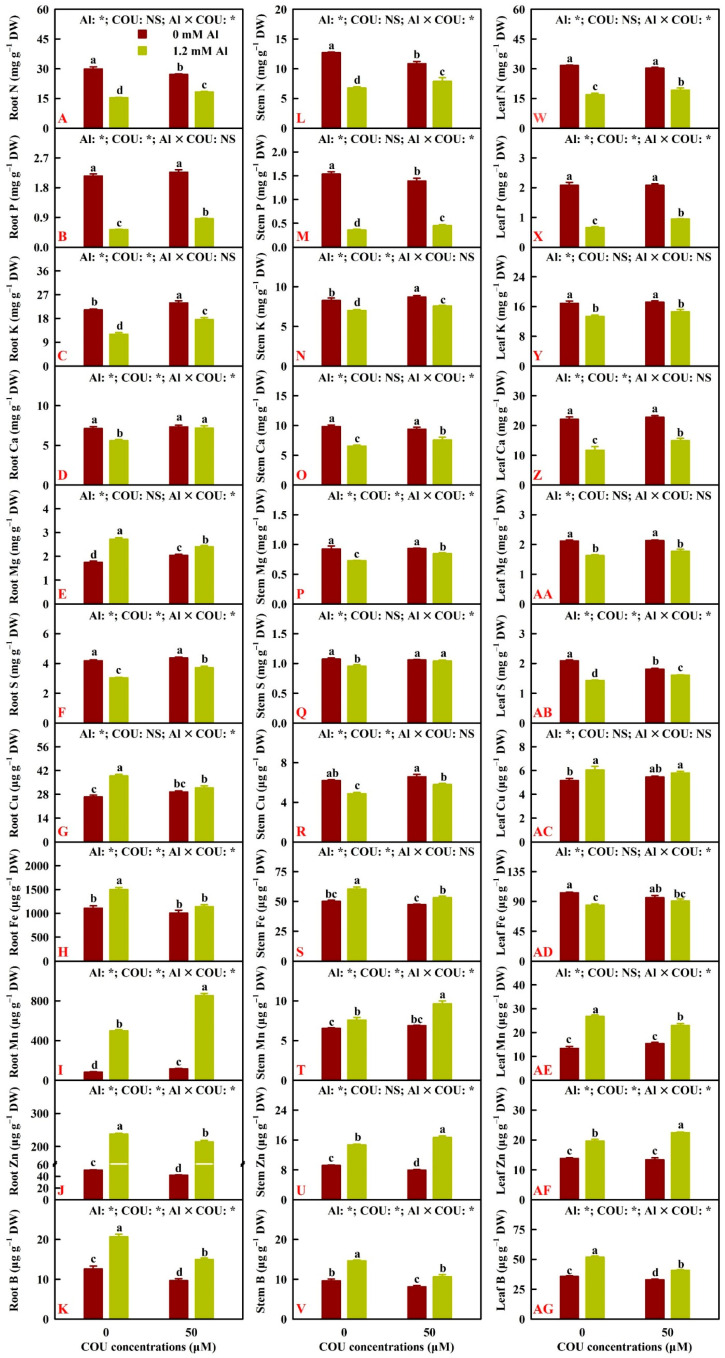
Effects of Al–COU treatments on the mean (±SE, *n* = 4) concentrations of 11 nutrients in roots (**A**–**K**), stems (**L**–**V**), and leaves (**W**–**AG**). Bars with different letters are significantly different at *p* ≤ 0.05. Al: *, COU: *, and Al × COU: * represent that the *F* values for Al, COU, and Al × COU are significant at *p* ≤ 0.05. HA: NS and Al × COU: NS represents that the *F* values for COU and Al × COU are not significant (*p* > 0.05).

Figure 6 displays the responses of leaf, root, and stem nutrient concentrations to Al–COU treatments. It was found that COU50 attenuated Al1.2-triggered reductions in root, leaf, and stem concentrations of Mg, K, S, P, N, and Ca, except that COU50 mitigated Al1.2-induced increase in root concentration of Mg. By contrast, COU50 alleviated Al1.2-triggered increases in root, leaf, and stem concentrations of boron (B). In general, COU50 reduced Al1.2-induced alterations in root, leaf, and stem concentrations of Zn, Fe, Cu, and Mn, except that COU50 promoted Al1.2-triggered increases in root and stem concentrations of Mn and in stem and leaf concentrations of Zn.

Figure S1 shows the responses of nutrient UPP and UPR to Al–COU treatments. It was observed that COU50 mitigated the decreases in Ca, K, P, N, S, Mg, and B UPP and UPR caused by Al.12 and the increases in Fe and Cu UPR caused by Al1.2, but it promoted the increases in Mn and Zn UPP and UPR caused by Al.2, except that COU50 did not significantly alter Zn UPR at Al1.2. Al^3+^ toxicity decreased Cu and Fe UPP, and COU50 significantly increased Cu UPP and did not significantly alter Fe UPP.

Figure S2 shows the effects of Al–COU treatments on the ratios of K, N, S, Ca, and Mg concentrations to P concentration in roots or leaves (hereafter referred to root or leaf K/P, N/P, S/P, Ca/P, and Mg/P) and the ratios of K, N, S, Ca, and Mg UPP to P UPP (hereafter referred to plant K/P, N/P, S/P, Ca/P, and Mg/P). Because Al1.2 decreased P concentrations in stems, roots, and leaves more than other macronutrient concentrations (Figure 6), Al1.2 caused a significant increase in root, leaf, and plant K/P, N/P, S/P, Ca/P, and Mg/P, with a lower increase at COU50 than at COU0.

Figure S3 shows the responses of nutrient distributions in roots, leaves, and stems to Al–COU treatments. The results showed that COU50 mitigated the increases of 11 nutrient distributions in roots caused by Al1.2, except that Mn distribution in roots at Al1.2 did not differ between the two COU treatments. In general, COU50 prevented the decreases of 11 nutrient distributions in leaves caused by Al1.2. It was observed that COU50 mitigated Al1.2-triggered decreases in N, Mg, Cu, Fe, Mn, and Zn distribution in stems, but promoted an Al1.2-triggered decrease in P distribution in stems; that Al1.2 significantly decreased K, S, and B distributions in stems at COU50, but not at COU0; and that Al1.2 did not significantly change Ca distribution in stems, but Ca distribution in stems at Al1.2 was lower at COU50 than at COU0.

### 2.4. Malondialdehyde, H_2_O_2_ Production Rate (HPR), Antioxidant Enzymes and Related Physiological Parameters in Roots and Leaves

It was shown that Al1.2 increased leaf HPR by 53.9% and 14.5%, root HPR by 33.5% and 26.8%, leaf MDA level by 64.1% and 23.0%, and root MDA level by 193.1% and 54.5% at COU0 and COU50, respectively, at *p* ≤ 0.05 (Figure S4A,B). Addition of COU prevented the decreases in root and leaf activities of ascorbate peroxidase (APX), superoxide dismutase (SOD), monodehydroascorbate reductase (MDHAR), guaiacol peroxidase (GuPX), and catalase (CAT) caused by Al1.2 (Figure S4C–G).

The results showed that Al1.2 significantly increased leaf total flavonoids level by 18.9% and 9.6%, root total flavonoids level by 51.5% and 32.5%, leaf DPPH scavenging activity by 31.4% and 13.8%, root DPPH scavenging activity by 21.1% and 15.3%, leaf TPs level by 52.8% and 21.5%, and root total COUs level by 12.3% and 11.8% at COU0 and COU50, respectively, as well as root TPs level by 45.1% at COU0 at *p* ≤ 0.05; however, it did not significantly alter root concentration of TPs at COU50 and leaf concentration of total COUs at COU0 and COU50 (Figure S4H–K).

### 2.5. Nonstructural Carbohydrates, TSPs, and TFAAs in Roots and Leaves

**Figure 7:**
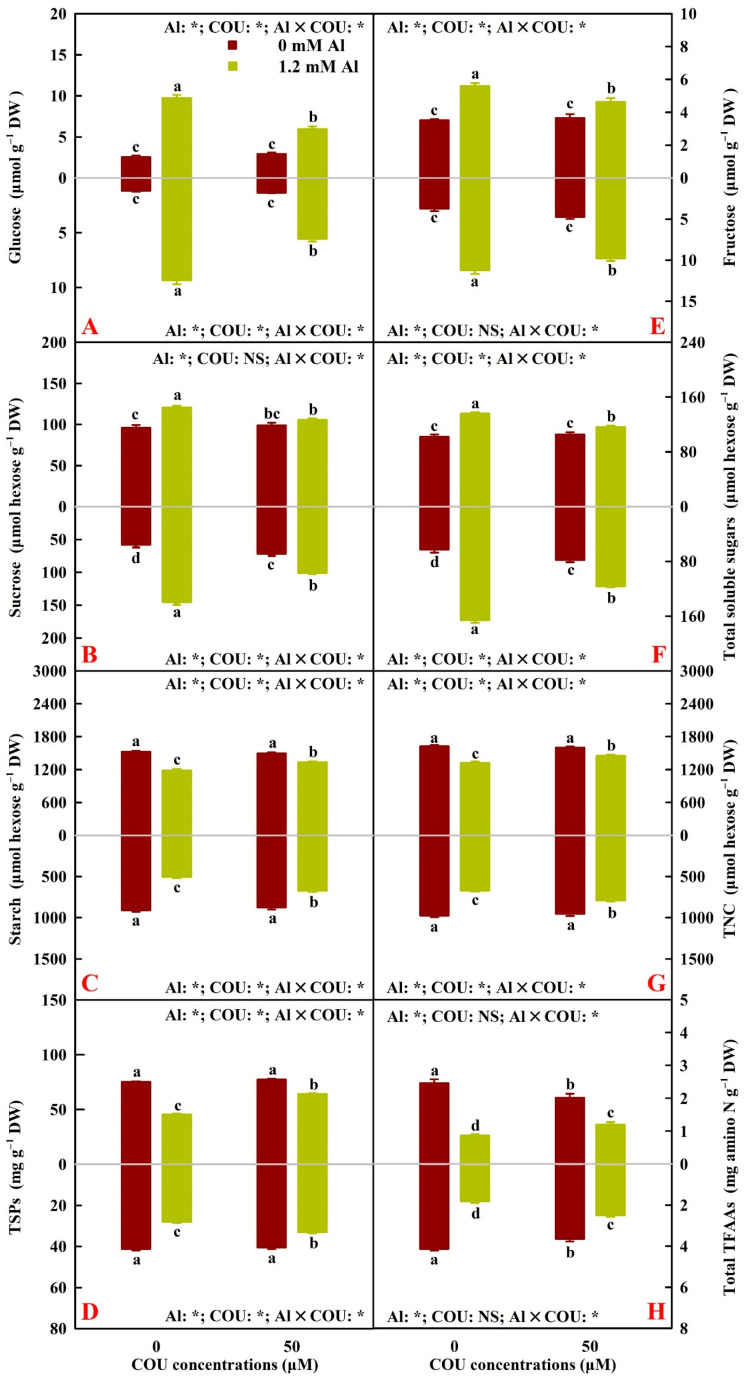
Mean (±SE, *n* = 4) concentrations of leaf (above column) and root (below column) NCs (**A**–**C**,**E**–**G**), TSPs (**D**), and TFAAs (**H**) in response to Al–COU treatments. Bars with different letters are statistically significant (*p ≤* 0.05). Al: *, COU: *, and Al × COU: * represent that the *F* values for Al, HA, and Al × HA are significant at *p* ≤ 0.05. COU: NS represents that the *F* value for COU is not significant (*p* > 0.05).

It was found that COU50 prevented Al1.2-induced increases in root and leaf concentrations of fructose (Fru), sucrose (Suc), glucose (Glu), and TSS (Fru + Suc + Glu), as well as decreases in root and leaf concentrations of starch and total NCs (TNC, TSS + starch) (Figure 7A–C,E–G). Addition of COU prevented the decreases of root and leaf concentration TSPs and TFAAs caused by Al1.2 (Figure 7D,H).

### 2.6. Principal Coordinate Analysis Plots and Differential Responses of Leaf and Root Parameters Ato Al–COU Treatments

**Figure 8:**
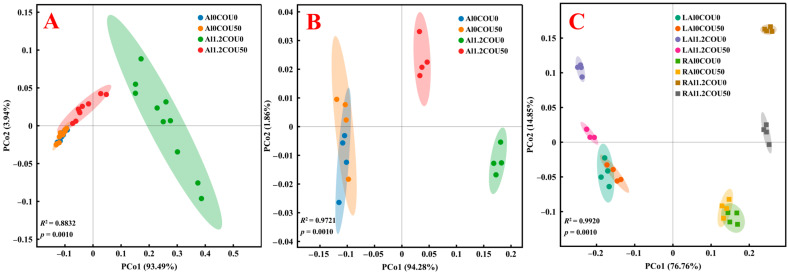
Principal coordinate analysis plots of 24 indexes for growth and fluorescence (**A**) and 158 other (**B**) indexes, as well as 48 common parameters present in both leaves and roots (**C**) from ‘Sour pummelo’ seedlings exposed to different Al–COU treatments. Al0COU0, Al0 + COU0; Al0COU50, Al0 + COU50; Al1.2COU0, Al1.2 + COU0; Al1.2COU50, Al1.2 + COU50; RAl0COU0, roots of Al0COU0-treated seedlings; RAl0COU50, roots of Al0COU50-treated seedlings; RAl1.2COU0, roots of Al1.2COU0-treated seedlings; RAl.2COU50, roots of Al1.2COU50-treated seedlings; LAl0COU0, leaves of Al0COU0-treated seedlings; LAl0COU50, leaves of Al0COU50-treated seedlings; LAl1.2COU0, leaves of Al1.2COU0-treated seedlings; LAl.2COU50, leaves of Al1.2COU50-treated seedlings.

**Figure 9:**
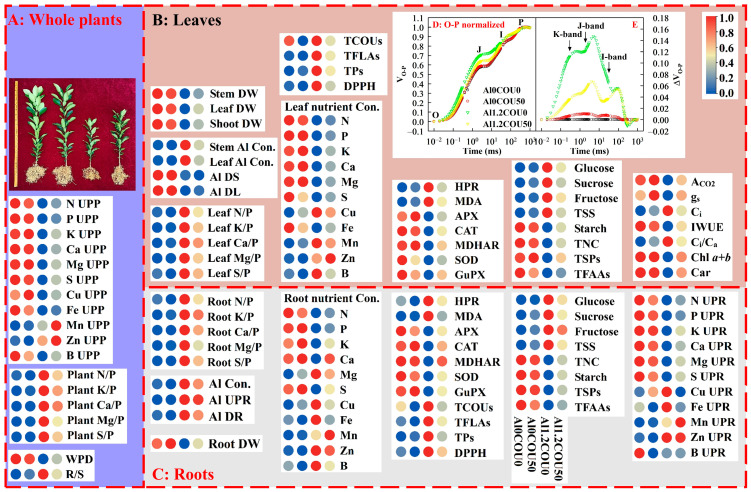
A diagram showing the effects of Al–COU treatments on growth, Al and nutrient uptake, and related physiological parameters in ‘Sour pummelo’ seedlings. Con., concentration; DL, distribution in leaves; DR, distribution in roots; DS, distribution in stems; TCOUs, total coumarins; TFLAs, total flavonoids; WPD, whole plant DW; (**A**) leaf parameters except for stem DW, stem Al Con., and Al DS; (**B**) seedling growth and whole plant parameters; (**C**) root parameters; (**D**,**E**) OJIP transients expressed as the kinetics of relative variable fluorescence: between F_o_ and F_m_: V_O-P_ = (F_t_ − F_o_)/(F_m_ − F_o_) (**D**) and the differences of the four samples to the reference sample treated with Al0COU0 (ΔV_O-P_, (**E**)).

Figure 8A and B shows the PCoA plots for 24 fluorescence and growth and 158 other parameters, respectively. The first two components (PCo1 and PCo2) explained 97.43% (93.49% for PCo1 and 3.94% for PCo2) and 96.14% (94.28% for PCo1 and 1.86% for PCo2) of the total variation for 24 and 158 parameters, respectively. The PCo1 revealed clear COU impacts at Al1.2 and Al^3+^-toxic impacts. It was observed that COU50 prevented these parameter changes caused by Al1.2, as revealed by the shorter distance between Al1.2COU50 and Al0COU50 than between Al1.2COU0 and Al0COU0. This was backed up by the data that Al1.2 did not significantly affect five (one from Figure S1, three from Figure S3, and one from Figure S4) and 24 (four from Figure 2, one from Figure 4, six from Figure 6, three from Figure S1, five from Figure S3, six from Figure S4, and one from Figure 7) out of 182 indexes at COU0 and COU50, respectively; and that Al1.2-induced changes in OJIP transients (Figure 3) and most parameters were more pronounced at COU0 than at COU50 (Figure 9).

Figure 8C shows the PCoA plot for 48 parameters present in both roots and leaves. The PCo1 and PCo2 contributed 76.76% and 14.85% of the total variation, and leaf and root parameters were clustered on the left and right side, respectively. This implied that there were vast differences in root and leaf responses to Al–COU treatments, which agreed with the data that 11 (Al, N, S, B, Ca, K, Mg, Cu, Mn, Zn, and Fe distribution in leaves) out of 48 leaf parameters exhibited a significant negative relation to the corresponding root parameters (Table S1). Additionally, Al1.2 increased the levels of Fe at COU0 and of Mg at COU0 and COU50 in roots, as well as P distribution in roots, but the reverse was the case in leaves (Figure 6 and Figure S3). Al1.2 significantly augmented the level of total COUs in roots, but not in leaves (Figure 4H). Notably, the PCoA showed that Al1.2 had a greater influence on the 48 parameters in roots than in leaves, as evidenced by the longer distance between RAl1.2COU0(50) and RA0COU0(50) than between LAl1.2COU0(50) and LA0COU0(50) (Figure 8C). This could be explained by the increased Al distribution in roots and far higher Al concentration in roots than in leaves at Al1.2 (Figure 5), as well as a greater increase in MDA concentration caused by Al1.2 in roots than in leaves (Figure S4B).

## 3. Discussion

### 3.1. Decreased Tissue Al Concentrations Rather than Augmented Al Distribution in Roots Played a Role in COU-Mediated Amelioration of Al^3+^ Toxicity in ‘Sour Pummelo’ Seedlings

The results clearly demonstrated that COU50 mitigated Al^3+^ toxicity in ‘Sour pummelo’ seedlings, as evidenced by the augmented growth at Al1.2 (Figure 1) and leaf A_CO2_ (Figure 2A). Cell wall (CW) is the main site for Al accumulation in plants [35,36]. In addition to acting as the main pool of Al accumulation, root CW is also the first physical barrier, hampering Al from entering more sensitive targets at the plasma membrane, symplasm, and/or shoots [37,38]. It was found that stem and leaf concentrations of Al under Al^3+^ toxicity were lower in Al-resistant ‘Xuegan’ seedlings than in Al-sensitive ‘Shantian pummelo’ ones, while root concentration of Al was similar between the two cultivars; and that both Al UPP and Al distribution in roots were higher in Al-resistant than in Al-sensitive cultivars under Al^3+^ toxicity [6]. Li et al. [36] reported that overexpression of *WRKY47* enhanced Al^3+^ tolerance of transgenic *A. thaliana* plants, while lack of WRKY47 reduced Al^3+^ tolerance; and that lack of WRKY47 led to increased accumulation of Al in root symplast and decreased accumulation of Al in apoplast. A plant’s tolerance to Al^3+^ toxicity is related to relatively lower root-to-shoot Al transport [39]. The research indicated that the mitigation of Al^3+^ toxicity mediated by COU50 was not due to lessened root-to-shoot Al transport, because COU50 did not affect Al distribution in roots with or without Al^3+^ toxicity (Figure 5). Notably, CW is also the target of Al^3+^ toxicity. Root growth inhibition caused by Al^3+^ toxicity is associated with the impairment of CW functions [40]. Less Al absorption is involved in a plant’s resilience to Al^3+^ toxicity [39]. The research showed that COU50 prevented Al1.2-triggered increases of tissue Al concentrations and Al UPR, and tissue Al concentrations and Al UPR at Al1.2 were lower at COU50 than at COU0 (Figure 5). Regression analysis indicated that any one of four indexes (root, leaf, and stem Al concentrations and Al UPR) was significantly negatively related with any one of whole plant, root, leaf, shoot, and stem DW (Table S1). Xu et al. [41] reported that methyl jasmonate (MJ, a phenolic synthesis precursor)-mediated mitigation of Al^3+^ toxicity in Chinese fir (*Cunninghamia lanceolata* (Lamb.) Hook) plants involved lower root Al concentration and less increase in root Al concentration under Al^3+^ toxicity relative to Al alone; and that 2-aminoindan-2-phosphonic acid (AIP, a phenolic synthesis inhibitor) significantly promoted Al^3+^ toxicity-induced inhibition of root growth accompanied by an increase in root Al concentration, but with no significant difference relative to Al alone. It was observed that COU-mediated mitigation of Mn toxicity in sesame plants involved lower root and leaf Mn concentrations and less increases in tissue Mn concentrations under Mn excess [16]; and that COU-mediated amelioration of Cu toxicity in ‘Xuegan’ seedlings involved lower tissue Cu concentrations and less increases in tissue Cu concentrations under Cu excess [15]. These results indicated that the COU50-mediated mitigation of Al^3+^ toxicity in ‘Sour pummelo’ seedlings was caused by the decrease in Al UPR and subsequent decrease in tissue Al concentrations rather than by augmented Al distribution in roots. Notably, regression analysis showed that root levels of TFAAs were significantly negatively related to Al UPR and root levels of Al (Table S1), and leaf levels of TFAAs were significantly negatively related to leaf levels of Al (Figure S1). Tang et al. [11] reported that γ-aminobutyric acid (GABA)-mediated mitigation of Al^3+^ toxicity in broad bean (*Vicia faba* L.) seedlings involved reduced concentrations of root and shoot Al. These results suggested that the lower reduction in the concentrations of root and leaf TFAAs mediated by COU50 (Figure 7) might contribute to the lower tissue Al concentrations at Al1.2.

### 3.2. Improved Nutrient Homeostasis Played a Role in COU-Mediated Amelioration of Al^3+^ Toxicity in ‘Sour Pummelo’ Seedlings

Al^3+^, which is detrimental to plant roots, inhibits root growth and function. This in turn interferes with water and nutrient absorption, thereby lowering crop growth and yield [3,42,43,44]. The current research showed that COU50 attenuated Al1.2-triggered decreases in tissue N, P, S, Mg, K, and Ca concentrations (Figure 6), as well as declines in their UPP and UPR (Figure 7). It was observed that root, leaf, and stem levels of N, P, S, Mg, K, and Ca were significantly positively related with the corresponding tissue DW or displayed an augmented trend with the increase in corresponding tissue DW, except for the relationship between root Mg level and root DW (*r* = −0.9031); and that any one of N, P, S, Mg, K, and Ca UPP and UPR was significantly positively related with any one of root, shoot, stem, leaf, and whole plant DW, except for root DW in relation to P UPP (*r* = 0.9420), N UPP (*r* = 0.9444), P UPR (*r* = 0.9356), N UPR (*r* = 0.9253), and S UPR (*r* = 0.9464) (Table S1). It was reported that the augmented leaf and root concentrations of N, P, Mg, K, Ca, Zn, and Fe played a role in COU-mediated mitigation of Mn excess in sesame plants [16]; and that the increased UPP and UPR of N, P, S, Mg, K, and Ca functioned in COU-mediated amelioration of Cu excess in ‘Xuegan’ seedlings [15]. There is evidence showing that Ca [45], Mg [46], S [14], and P [43] can attenuate the growth reduction caused by Al^3+^ toxicity. Phosphorus deficiency has been suggested to be the cause for the Al^3+^-induced growth decline of triticale (× *Triticosecale* Wittmack) seedlings [47]. The amelioration of Al^3+^ toxicity in white clover (*Trifolium repens* L.) plants mediated by arbuscular mycorrhiza involved strengthened P acquisition [48]. In sorghum (*Sorghum bicolor* L.) plants, Al^3+^-inhibited root development and lessened absorption of N, Mg, and K may partly account for the growth decline. Supplementation of P plays a certain role in mitigating A1^3+^ toxicity, possibly by improving root development and augmenting nutrient absorption [49]. Augmented absorption of P, Ca, and Mg exerts a role in S-mediated amelioration of Al^3+^ toxicity in barley (*Hordeum vulgare* L.) seedlings [14]. Cell expansion can be disturbed by Al^3+^ via the displacement of Ca^2+^ [50]. Mitigation of Ca-mediated Al^3+^ toxicity in plants involves the displacement of cell surface Al^3+^ by Ca^2+^ and the restoration of Ca^2+^ at the cell surface [46]. As osmolytes, metal chelators, antioxidants, the building blocks for protein synthesis, and the precursors for the various metabolite synthesis, such as phytohormones, amino acids (AAs) exert a key role in plant growth and stress resilience, such as oxidative and metal tolerance [16,51,52,53]. It was reported that N deficiency led to a significant decrease in leaf and root concentrations of N, TSPs, and TFAAs [54], as well as in root, leaf, stem, shoot, and whole plant DW in ‘Xuegan’ seedlings [55]; and that N deficiency caused a significant decrease in barley shoot and root concentrations of TSPs and TFAAs, as well as in shoot DW, but it did not significantly alter root DW [56]. Linear regression analysis showed that leaf and root levels of TSPs and TFAAs were significantly positively related with the corresponding tissue concentration of N, except for leaf N level in relation to TSPs concentration (*r* = 0.9037); and that leaf and root DW were significantly positively related with the corresponding tissue levels of TSPs and TFAAs, except for root DW in relation to TSPs level (*r* = 0.9349) (Table S1). These results suggest that COU50 prevented Al1.2-induced decreases in N absorption and tissue concentrations of N, TSPs, and TFAAs, thereby exerting a certain role in improving seedling Al tolerance and promoting seedling growth at Al1.2 (Figure 1). Notably, plants under stressed conditions often suffer from an energy deficit. The greater reductions in tissue concentrations of TSPs and TFAAs caused by Al1.2 at COU0 than at COU50 (Figure 7) agree with an increased demand for energy, since both AA and protein synthesis require a significant amount of energy consumption. Collectively, these results indicated that the augmented absorption of macronutrients (Mg, K, Ca, S, P, and N) exerts a certain role in COU-mediated mitigation of growth inhibition caused by Al1.2 (Figure 1).

The current results indicated that Al1.2 caused a significant increase in leaf, root, and plant S/P, N/P, Mg/P, K/P, and Ca/P, with a greater increase at COU0 than at COU50 (Figure S2), because Al1.2 caused a greater decrease in the UPP, UPR, and tissue concentration of P than in the UPP, UPR, and tissue concentrations of S, N, Mg, K, and Ca, especially at COU0 (Figure 6 and Figure S1). The study revealed a significant positive correlation between any one of the five parameters (root, leaf stem, shoot, and whole plant DW) and any 1 of the 15 parameters (root, leaf, and plant S/P, K/P, N/P, Ca/P, and Mg/P) (Table S1). Nutrient imbalance in plants can limit their growth [57,58]. The molecular responses in chickpea (*Cicer arietinum* L.) leaves and roots induced by nitrate or P imbalance were more pronounced than those induced by combined nitrate and P starvation [59]. These results indicated that enhanced ability to absorb P and to maintain the balance of macronutrients exerted a certain role in COU-mediated amelioration of growth inhibition caused by Al1.2 (Figure 1).

It was observed that Al^3+^ toxicity augmented R/S (Figure 1), as observed on peach (*Prunus persica* L.) [60]. However, Al^3+^ toxicity did not alter R/S in ‘Swingle’ citrumelo (*Citrus paradisi* Mcf. × *P. trifoliata*) plants [61]. Guo et al. [6] observed that 1 mM AlCl_3_ increased R/S in Al-sensitive ‘Shantian pummelo’ seedlings, but not in Al-resistant ‘Xuegan’ seedlings. Tóth et al. [62] found that Al^3+^ toxicity caused a decrease in R/S of 23 out of 25 common bean cultivars, but it did not affect the R/S of the other two cultivars. These results suggested that the impact of Al^3+^ on R/S depended on plant species and/or cultivars.

It was observed that Al1.2 caused a significant increase in the distributions of all 11 elements in roots at COU0 and COU50, except for the distribution of K in roots at COU50 (Figure S3), and that R/S displayed a significant linear increase with increasing B, N, P, S, Ca, Cu, Fe, K, and Mg distributions in roots and an increased trend with increasing Mn and Zn distributions in roots (Table S1). The increased distributions of nutrients in roots caused by Al1.2 at least partially explained the increase in R/S at Al1.2. Plant root systems are strongly affected by nutrient availability [63]. It is known that nutrient (N, K, Ca, S, Mg, and P) starvation can cause an increase in R/S in various plants, including arable crops [63], *Spathiphyllum* spp. [64], *Citrus* [55], wheat, pea (*Pisum sativum* L.), and common bean (*Phaseolus vulgaris* L.) [65]. Notably, Al1.2 significantly decreased the N, K, Ca, S, Mg, and P UPP; and R/S displayed a significant linear increase with decreasing N, K, Ca, S, Mg, and P UPP (Table S1). This suggested that the decreased uptake of nutrients might contribute to the increased R/S at Al1.2. This was supported by the view that the increased R/S under nutrient-poor conditions facilitates the exploration of nutrient-rich patches or compensates for the deficiencies in nutrients underground [66]. These results indicated that the increment in R/S caused by Al1.2 was an adaptive strategy that provides nutrients to relatively fewer shoots through relatively more roots.

To conclude, COU50 endowed seedlings with the ability to maintain nutrient (P, N, S, Ca, K, and Mg) homeostasis at Al1.2, thus promoting their growth.

### 3.3. Reduced Oxidative Damage Played a Role in COU-Mediated Amelioration of Al^3+^ Toxicity in ‘Sour Pummelo’ Seedlings

It is known that Al^3+^ can stimulate the formation of excessive ROS, thereby inducing oxidative impairment to cellular components [67,68]. Because Al1.2 affected leaf A_CO2_ more at COU0 than at COU50 (Figure 2A), the amount of excess absorbed photo flux density (PFD) was higher in LAl1.2 than in leaves of Al0-treated seedlings (LAl0), especially at COU0. The excess absorbed PFD can trigger the formation of ROS [69]. The results indicated that in addition to decreasing Al UPR (Figure 5H), COU50 attenuated Al1.2-induced increases in leaf and root levels of Al (Figure 5A,C), HPR and MDA (Figure S4A,B), and decreases in leaf and root concentrations of TSPs (Figure 7D) and in leaf A_CO2_ (Figure 2A). It was found that Al UPR was significantly positively related to root (Table S1) and leaf Al (Figure S1) concentrations. For leaves or roots, HPR was significantly positively related to Al and MDA concentrations, and TSPs concentration was significantly negatively related to MDA concentration (Table S1 and Figure S5). For leaves, HPR was significantly negatively related to A_CO2_ (Table S1). These results suggested that COU50 attenuated Al1.2-triggered increases in Al UPR and leaf and root Al concentrations and a decrease in leaf A_CO2_, thereby lowering ROS formation and oxidative stress in leaves and roots, and preventing oxidative stress-induced decrease in leaf and root concentrations of TSPs at Al1.2.

Al^3+^-triggered oxidative stress has been suggested as one of the main causes of Al^3+^ toxicity [70,71]. The enzymatic antioxidant system plays a vital role in the ROS homeostasis of higher plants [67,72]. O_2_**^·^**^−^ is often the first ROS generated in plant tissue and can be dismutated into H_2_O_2_ by SOD. The yielded H_2_O_2_ can be reduced to H_2_O by CAT, APX, and/or GuPX. Monodehydroascorbate reductase plays a key role in plant’s tolerance to oxidative stress by converting monodehydroascorbate, produced by APX, back into ascorbate (ASC) [73,74]. It was found that COU50 prevented Al1.2-induced decreases in root and leaf activities of five antioxidant enzymes (Figure S4C–G), and that leaf and root concentrations of MDA were significantly negatively related with the corresponding tissue activities of APX, MDHAR, SOD, CAT, and GuPX (Table S1 and Figure S5). A study showed that Al-tolerant ‘Xuegan’ leaves and roots kept higher antioxidant enzyme activities than Al-sensitive ‘Shatian pummelo’ leaves and roots did in response to Al^3+^ toxicity [6]. Similar results have been observed in popcorn (*Zea mays* L.) [74], sorghum [75], and rice [68]. There was a study showing that the upregulation of genes involved in ROS detoxifying in response to Al^3+^ toxicity was greater in ‘Xuegan’ roots than in ‘Shatian pummelo’ roots [76]. Upregulation of antioxidant enzyme genes enhanced the tolerance of transgenic tobacco (*Nicotiana tabacum* L.) plants overexpressing an *A. thaliana* peroxidase gene (*AtPrx64*) [77] and rapeseed (*Brassica napus* L.) plants overexpressing a wheat *WMnSOD1* [78] to Al^3+^ toxicity. Previous studies indicated that B-mediated amelioration of oxidative injury caused by Al^3+^ in trifoliate orange seedlings [79], Mg-mediated amelioration of oxidative injury caused by Al^3+^ in apple rootstock M26 (*M. domestica*) plants [46], and S-mediated amelioration of oxidative injury caused by Al^3+^ in ‘Shatian pummelo’ seedlings [80] involved enhanced activities of antioxidant enzymes. Shad et al. [16] found that COU-mediated alleviation of oxidative injury caused by Mn excess in sesame plants involved augmented activities of MDHAR, SOD, APX, CAT, and GuPX. These results suggested that increased activities of antioxidant enzymes functioned in COU-mediated mitigation of oxidative damage at Al1.2.

In addition to antioxidant enzymes, non-enzymatic antioxidants (AAs, soluble sugars, and phenolic compounds) can also scavenge ROS and lessen oxidative damage in higher plants under stress conditions [16,41,81]. Phenolic compounds, the pivotal SMs in plants, display strong antioxidant activity [41,82]. The accumulation of phenolic compounds in woody plant roots was positively related to their Al tolerance [20]. Chen et al. [82] observed significant increases in TP concentration and DPPH scavenging activity in Al-tolerant lettuce (*Lactuca sativa* L.) roots caused by Al^3+^ toxicity. Increased accumulation of phenolic compounds was able to protect plants from oxidative stress caused by Al^3+^ toxicity. Xu et al. [41] observed that MJ (a phenolic synthesis precursor)-mediated mitigation of oxidative damage in Chinese fir plants caused by Al^3+^ toxicity involved decreased accumulation of ROS and increased activities of key enzymes related to phenolic metabolism and antioxidant enzymes, as well as increased DPPH scavenging activity. They concluded that phenolic compounds functioned in the Al tolerance of Chinese fir. The antioxidant capacity of ellagic acid (EA, a phenolic compound) involves its ROS detoxifying capacity, induction of cellular antioxidant enzyme activity, and metal-chelating ability. It was observed that EA-mediated mitigation of oxidative damage in maize plants involved reduced accumulation of ROS and increased activities of antioxidant enzymes [12]. Flavonoids are a large class of phenolic compounds. Stress-responsive dihydroxy B-ring-substituted flavonoids in plants are effective scavengers of ROS [83]. It was suggested that flavonoid-type phenolics played a role in silicon (Si)-mediated amelioration of Al^3+^ toxicity in maize seedlings [84]. The antioxidant effects of COUs include inhibition of ROS-generating enzymes, chelation of metals, and/or detoxification of ROS [85]. Amino acids can help plants better tolerate oxidative damage [16]. γ-Aminobutyric acid-mediated mitigation of oxidative damage in creeping bentgrass (*Agrostis stolonifera* L.) plants caused by Al^3+^ toxicity involved increased accumulation of AAs and sugars, elevated activities of antioxidant enzymes, and decreased accumulation of ROS [86]. Soluble sugars play a role in safeguarding plants from oxidative injury [81,87]. The high Al tolerance of ‘Duke’ (*Vaccinium corymbosum* L.) plants was associated with increased antioxidant activity, high concentrations of soluble sugars and total phenols, and low lipid peroxidation in leaves [88]. Shad et al. [16] indicated that COU-mediated amelioration of oxidative stress in sesame seedlings caused by excessive Mn involved decreased accumulation of ROS, enhanced accumulation of TPs, reducing sugars, TSS, total flavonoids, and TFAAs, and increased DPPH scavenging activity. Diphenylpicrylhydrazyl scavenging activity has been widely used to evaluate the antioxidant activity of compounds (extracts) [6,89,90]. It was found that among four extracts of *Pholidota chinensis* Lindl., the ethyl acetate extract not only had the highest concentration of TPs but also possessed the strongest DPPH scavenging activity (antioxidant activity); and that the antioxidant activity was significantly positively correlated with the concentration of TPs [90]. The research showed that COU50 attenuated Al1.2-induced increases in root and leaf DPPH scavenging activity, root and leaf concentrations of soluble sugars, TPs, and total flavonoids, and root concentration of total COUs, and decreases in root and leaf concentrations of TFAAs (Figure 7 and Figure S4). Regression analysis showed that for leaves or roots, any one of HPR, MDA, and DPPH was significantly positively related with any one of total flavonoids, TPs, Glu, Suc, Fru, TSS, and DPPH and negatively related with TFAAs (Table S1 and Figure S5), except for root HPR in relation to TPs (*r* = 0.9400), Suc (*r* = 0.9164), TSS (*r* = 0.9206), and TFAAs (*r* = −0.8743) (Table S1); that for leaves or roots, DPPH was significantly positively related with HPR and MDA; and that root concentration of total COUs increased with increasing HPR (*r* = 0.9168), MDA concentration (*r* = 0.9287), and DPPH scavenging activity (*r* = 0.8325) (Table S1). These results indicated that Al1.2-induced upregulation in phenolic compounds, total flavonoids, TSS, total COUs, and DPPH scavenging activity was an adaptive response to Al1.2, but this did not explain the COU-mediated mitigation of oxidative stress caused by Al1.2, because the upregulation was more pronounced at COU0 than at COU50. The greater upregulation of these non-enzymic antioxidants and DPPH caused by Al1.2 at COU0 agreed with the augmented demand for ROS scavenging (Figure S4A). The lower decrease in root and leaf levels of TFAAs caused by Al1.2 at COU50 might contribute to the reduced oxidative damage caused by Al1.2.

Excessive accumulation of ROS caused by Al^3+^ toxicity can oxidize biomolecules like nucleic acids, proteins (enzymes), and lipids, eventually leading to plant cell death and biomass reduction [67,91]. Linear regression analysis showed that for roots or leaves, the concentration of TSPs was significantly negatively related to HPR and the concentration of MDA, and that leaf or root DW was significantly negatively related to the corresponding tissue levels of HRP, MDA, and TSPs (Figure S5 and Table S1). These results indicated that COU50 reduced the oxidation of biomolecules (proteins) via reducing the production and over-accumulation of ROS, thereby preventing Al1.2-induced inhibition of seedling growth (Figure 1).

To conclude, COU50 endowed roots and leaves with the capacity to relieve oxidative damage caused by Al1.2 through reducing ROS production, upregulating antioxidant enzyme activities, and keeping the homeostasis of non-enzymatic antioxidants under Al^3+^ toxicity.

### 3.4. Reduced Tissue Al Concentration and Enhanced Capability to Maintain Nutrient and Redox Homeostasis Played a Role in COU-Mediated Mitigation of Leaf A_CO2_ Decline at Al1.2

The increases in both C_i_ and C_i_/C_a_ in response to Al1.2 suggested that the decline in leaf A_CO2_ at Al1.2 was primarily attributed to non-stomatal limitation rather than stomatal limitation despite a significant decrease in g_s_ (Figure 2). Similar results have been reported on *Citrus* [4], common bean [92], and sorghum [93]. A significant positive relationship between A_CO2_ and any one of Chl *a+b*, Chl *a*, and Chl *b* (Table S1) did not necessarily mean that the decline in leaf A_CO2_ in response to Al1.2 was caused by decreased Chl concentrations, because A_CO2_ decreased more than Chl concentrations in response to Al1.2. This agreed with the results that the increase in DI_o_/RC caused by Al1.2 was lower at COU50 than at COU0 (Figure 4J), and there was a significant negative relationship between DI_o_/RC and A_CO2_ (Table S1). Also, a greater reduction in A_CO2_ than in Chl level in response to Al^3+^ toxicity has been obtained on leaves of Al-treated *Eucalyptus* spp. [50], common bean [92], ‘Cleopatra’ tangerine (*Citrus reshni* Hort. ex Tanaka) [94], and Xuegan’ [95] plants.

High concentration of Al^3+^ can cause Chl breakdown and impair Chl biosynthesis [96,97]. Linear regression analysis showed that a significant negative relationship existed between leaf Al concentration and any one of F_v_/F_o_, Chl *a*, Chl *b*, and Chl *a+b* (Table S1), suggesting that COU50 attenuated Al1.2-triggered increment in Chl degradation and decrease in Chl biosynthesis due to reduced Al concentration, thereby improving Chl concentration at Al1.2. The replacement of Mg, the central atom of a Chl molecule, by Al can cause a decrease in Chl concentration [96]. The research showed that COU50 attenuated the decrease in leaf Mg concentration (Figure 6A) and the increase in leaf Al concentration (Figure 5C) caused by Al1.2; that any one of Chl *a*, Chl *b*, and Chl *a+b* increased significantly with elevating leaf Mg concentration in a curvilinear manner (Figure S5); and that a significant positive relationship existed between F_v_/F_o_ and any one of leaf Mg concentration, Chl *a*, Chl *b*, and Chl *a+b* (Table S1). These results indicated that reduced replacement of Mg by Al played a role in the COU-mediated mitigation of Chl decline and thylakoid damage caused by Al1.2. Ali et al. [98] indicated that H_2_S might attenuate Al^3+^ toxicity-induced destruction of chloroplast thylakoids in rapeseed, thereby alleviating Chl decline under Al^3+^ toxicity. Also, exogenous application of S [80] and P [99] mitigated the decrease in leaf Chl concentration and the impairment of thylakoid structure caused by Al^3+^ toxicity. Recently, Vasconcelos et al. [92] indicated that Ca attenuated the decrease in the Chl concentration in common bean leaves caused by Al^3+^ toxicity. There was a study showing that in K- and N-deficient rapeseed leaves, disruption of chloroplast structure was synchronous with the Chl loss [100]. The current research showed that Chl *a*, Chl *b*, Chl *a+b*, or F_v_/F_o_ significantly increased with increasing foliar concentration of N, P, Ca, K, or S in a linear (Table S1) or curvilinear (Figures S5 and S6) manner except for leaf N concentration in relation to Chl *a* (*r* = 0.8316), Chl *b* (*r* = 0.8327), and Chl *a+b* (*r* = 0.8271) (Table S1). These results suggested that COU50 attenuated Al1.2-triggered increase in foliar level of Al and decreases in foliar levels of N, P, S, Ca, K, and Mg, thereby mitigating the damage of Al1.2 to thylakoids and subsequently resisting Chl decline.

It is known that photooxidative damage can destroy chloroplast ultrastructure and lead to pigment bleaching and death [101]. It was shown that there was a significant negative relationship between any one of leaf HRP and MDA and any one of Chl *a+b*, Chl *a*, Chl *b*, or F_v_/F_o_ (Table S1). These results indicated that COU-mediated mitigation of leaf oxidative stress caused by Al1.2 also played a role in preventing Al1.2-induced chloroplast ultrastructure damage and subsequent decline in Chl concentration.

The current findings that there was a significant negative relationship between A_CO2_ and any of leaf Glu, Fru, Suc, and TSS (Table S1) did not necessarily imply that feedback inhibition was responsible for leaf A_CO2_ decline caused by Al1.2, because the concentrations of leaf starch and TNC significantly decreased in response to Al1.2 (Figure 7). This agreed with the reports that in rye (*Secale cereale* L.) leaves, Al^3+^ toxicity increased and decreased the concentrations of soluble sugars and starch, respectively [102]; and in ‘Xuegan’ leaves and roots, Al^3+^ toxicity increased the concentrations of soluble sugars, but decreased the concentrations of starch and TNC [103]. When exposed to stressed conditions such as metal toxicity, plant starch (an energy storage molecule) can be rapidly hydrolyzed to soluble sugars to help plants mitigate oxidative stress by providing energy and serving as antioxidants [102,104]. Therefore, the increases in the levels of soluble sugars and the hydrolysis of starch in leaves and roots in response to Al1.2 might be an adaptation. However, COU-mediated mitigation of Al^3+^ toxicity could not be explained in this way. The greater increases in the levels of soluble sugars and the hydrolysis of starch in leaves and roots caused by Al1.2 at COU0 than at COU50 agreed with the elevated need for energy and ROS scavenging.

Excessive formation of ROS in chloroplasts can lead to a decrease in leaf photosynthesis via impairing chloroplast ultrastructure and function [105,106]. It was found that there was a significant negative relationship between any one of F_v_/F_o_ and A_CO2_ and any one of leaf MDA concentration and HPR, as well as a significant positive correlation between A_CO2_ and F_v_/F_o_ (Table S1). Taken together, COU50 attenuated Al1.2-induced leaf oxidative stress, thereby maintaining leaf thylakoid structure and function and subsequently improving A_CO2_ at Al1.2 (Figure 2A).

The replacement of Mg by Al can inhibit photosynthetic electron transport [96]. A study of wheat seedlings indicated that the reduction in photosynthesis in response to Al^3+^ toxicity was caused by an increase in the closure of PSII RCs and a decrease in the electron transport rate in PSII [107]. There are studies from *Citrus* showing that the damage of the whole PETC from PSII donor side to the reduction of photosystem I (PSI) was the main contributor to leaf A_CO2_ decline at Al^3+^ toxicity [95,108]. It was observed that COU50 attenuated Al1.2-triggered decreases in F_v_/F_m_ and ET_o_/ABS, an increment in DI_o_/RC (Figure 4), and changes in OJIP transients (Figure 3), implying that Al1.2-induced photoinhibition of PSII was lessened by COU50 [109]. It was shown that A_CO2_ was significantly negatively related with any one of M_o_, DI_o_/RC, V_J_, V_I_, and ABS/RC, and TR_o_/RC, and positively related with any one of F_v_/F_o_, F_m_, F_v_, F_v_/F_m_, RE_o_/ABS, PI_abs,total_, ET_o_/ABS, ET_o_/RC, RE_o_/RC, ET_o_/TR_o_, and MAIP; however, the reverse was the case for the relationships between leaf Al concentration and these fluorescence parameters (Table S1). It is known that both nutrient deficiencies and imbalances can damage the PETC and subsequently lower A_CO2_ [110,111,112]. The research showed that any 1 of 12 indexes (S, K, Ca, P, N, and Mg UPP and their concentrations in leaves) were significantly negatively related with any one of three parameters (M_o_, V_J_, and V_I_) and positively related with any one of ten parameters (A_CO2_, ET_o_/RC, RE_o_/RC, ET_o_/ABS, RE_o_/ABS, F_v_/F_o_, ET_o_/TR_o_, MAIP, F_v_/F_m_, and PI_abs,total_) with a few exceptions; however, the reverse was the case for the relationships between five ratio parameters (leaf S/P, N/P, Mg/P, K/P, and Ca/P) and these 13 parameters (Table S1 and Figures S5–S7).

Taken together, COU50 reduced tissue Al concentration and enhanced the ability of seedlings to maintain nutrient and redox homeostasis at Al1.2, thereby attenuating Al1.2-induced impairment of chloroplast structure and function and PETC and subsequently improving A_CO2_ at Al1.2.

### 3.5. The Differences in the Mitigation of COU to Plant Metal Toxicities

Previous and current studies indicated that exogenous application of COU alleviated the toxicity of various metals (Cu, Mn, Cr, Ni, and Al) to plants by reducing these metal concentrations and oxidative damage and enhancing nutrient (N, P, K, Ca, and Mg) concentrations in leaves and roots [13,15,16] (Figure 5 and Figure 6 and Figure S4). However, it was found that the distribution of Cu in roots decreased with the increase in COU supply, while the reverse was the case for the distribution of Cu in stems and leaves [15]; but addition of COU had no significant influence on the Al distribution in roots, stems, and leaves with the exception that the Al distribution in stems at Al1.2 was lower at COU50 than at COU0 (Figure 5). Notably, Al1.2 (Cu excess) increased the distribution of Al (Cu) in roots and reduced the Al (Cu) distribution in stems and leaves [15] (Figure 5). Shad et al. [16] reported that COU seed priming reduced the aerial translocation of Mn, thus protecting sesame plants from Mn toxicity. Ashraf et al. [13] showed that in castor bean seedlings, foliar application of COU prevented Cu excess-induced decrease in Cu translocation factor (*T_f_*), but COU–Ni (COU–Cr) treatments had no significant influence on the Ni (Cr) *T_f_*. It was shown that the addition of COU mitigated Cu excess-induced decrease in leaf and root concentration of B, and Cu–COU treatments did not alter the stem concentration of B [16], but the addition of COU alleviated Al1.2-induced increase in root, stem, and leaf concentration of B (Figure 6). The current study indicated that COU50 mitigated Al1.2-triggered increase in leaf and root concentrations of TPs and total flavonoids, as well as leaf and root DPPH scavenging activity (Figure S4). However, seed COU priming mitigated Mn excess-induced decrease in leaf DPPH scavenging activity, and the foliar concentrations of TPs and total flavonoids under Mn excess were not lower under seed COU priming than under seed hydro-priming [16]. Similarly, foliar application of COU prevented Cr-, Cu-, and Ni-induced decrease in leaf DPPH scavenging activity and increased or did not significantly alter the foliar concentrations of TPs and total flavonoids under these metal stress [13]. These findings indicated that the mechanisms by which COU alleviated the toxicity of different metals to plants were not the same.

## 4. Materials and Methods

### 4.1. Plant Materials

Seedling culture and Al–COU treatments were carried out as given by Huang et al. [15] and Guo et al. [80]. Briefly, the seeds of ‘Sour pummelo’ (*Citrus grandis* (L.) Osbeck) were sown in plastic trays filled with sand. After six weeks of germination, seedlings of uniform size were transported into 6 L flowerpots (two plants flowerpot^−1^) filled with sand, then cultivated in a greenhouse under natural conditions at Fujian Agriculture and Forestry University, Fuzhou (26°5′ N, 119°14′), with an annual average sunshine hours of ∼1600 h, temperature of ∼20 °C, and relative humidity of ∼76% [95]. After seven weeks of transplantation, each flowerpot was supplied six times per week with freshly prepared nutrition solution containing 1 mM Ca(NO_3_)_2_, 0.5 mM MgSO_4_, 0.1 mM KH_2_PO_4_, 1 mM KNO_3_, 10 μM H_3_BO_3_, 20 μM Fe-EDTA, 2 μM MnCl_2_, 0.5 μM CuSO_4_, 0.065 μM (NH_4_)_6_Mo_7_O_24_, 2 μM ZnSO_4_, 0 (COU0) or 50 (COU50) μM COU, and 0 (Al0) or 1.2 (Al1.2 or Al^3+^ toxicity) mM AlCl_3_·6H_2_O [15,80] until the sand was saturated (∼500 mL). The nutrient solution pH was adjusted to 4.2 with NaOH or HCl. There were four treatments, each with 12 flowerpots (a total of 48 flowerpots) arranged in a completely randomized design. After 18 weeks of Al–COU treatments, ~0.5-cm-long root apices and recently fully expanded leaves were used for all assays except for Al and nutrients. After leaf OJIP transient and gas exchange measurements, root apices and leaf discs with a diameter of 5 mm (one plant flowerpot^−1^) were collected and frozen in liquid N_2_ and then stored at −80 °C until they were used for the measurements of DPPH scavenging activity, enzyme activities, and metabolite concentrations. Seedlings from the same flowerpots without taken root apices and leaves were used to determine HPR, Al, nutrients, and biomass. In this study, the concentrations of AlCl_3_·6H_2_O and COU and the treatment duration were chosen according to previous studies [15,18,19,80] and our preliminary experiment. During our preliminary experiment, we investigated the impacts of three COU levels (0, 25, and 50 μM COU) and two Al levels (0 and 1.2 mM AlCl_3_·6H_2_O) on the growth of ‘Sour pummelo’ seedlings. Since 25 μM COU exhibited poor mitigation effects against Al^3+^ toxicity, we chose 0 and 50 μM COU for this study.

### 4.2. Chemicals

All chemicals used were analytical reagents or the best commercially available grade. Coumarin (CAS: 91-64-5; molecular formula: C_9_H_6_O_2_; MW: 146.14; analytical reagent) was purchased from Shanghai Aladdin Biochemical Technology Co., Ltd., Shanghai, China.

### 4.3. Gas Exchange and OJIP Transient Measurements

Leaf gas exchange was measured with a CIRAS-2 portable photosynthesis system (PP Systems, Herts, UK) between 9:30 and 11:00 a.m. at a controlled light intensity of ~1200 μmol m^−2^ s^−1^, a leaf temperature of ~22 °C, and a CO_2_ concentration of ~430 μmol mol^−1^ [80]. Instantaneous water use efficiency was calculated as follows: IWUE (μmol CO_2_ mmol^−1^ H_2_O) = A_CO2_/transpiration rate (T_r_).

Leaf OJIP transient was determined with a Handy PEA (Hansatech Instruments Limited, King’s Lynn, Norfolk, UK) after plants were dark-adapted at ~25 °C for 3 h [108]. Details of the JIP-test indexes are listed in Table S2 [108,111].

### 4.4. Measurements of Biomass, Al, Nutrients, and Pigments

Dry weights of roots, leaves, and stems were measured after they were dried at ~70 °C to a constant weight [15].

The middle sections of stems, the fibrous roots, and the recently fully expanded mature leaves were harvested, dried at 60 °C to a constant weight (~48 h), ground to pass a 40-mesh sieve, and then stored for analysis. The extraction and measurements of S, P, N, B, Zn, Mn, Mg, K, Fe, Cu, and Ca were carried out according to Huang et al. [15]. Aluminum was measured using aluminon [113] with some modifications. Briefly, 0.2 g of root, stem, and leaf powders were digested with a 5 mL mixture of HNO_3_:HClO_4_ (5:1, *v*:*v*). The yielded leaf and stem (root) digested solution was diluted to 50 (250, value in parentheses corresponds to root) mL with ultrapure water, filtered through filter paper, and used for the determination of Al. Zero point nine milliliter of digested solution was transferred to a 5 mL tube containing 1.8 mL of ultrapure water, then one drop of indicator (2 g of 4-nitrophenol dissolved in 1 L of ethanol) was added to the tube, followed by adjusting to yellow with 2 M NaOH and colorless with 0.5 M H_2_SO_4_. Thereafter, 0.18 mL of 1 g L^−1^ hydroxylamine hydrochloride was added to the tube and shaken well. After 5 min, 0.05 mL of 0.5 M ammonium acetate-acetic acid buffer (pH 4.5) was added to the tube and shaken well, followed by adding 0.18 mL of 1 g L^−1^ aluminon and mixing. After 30 min, the color intensity of the solution was measured at 520 nm with a UV spectrophotometer (Libra S22, Biochrom Ltd., Cambridge, UK). The calculation of nutrient uptake and distribution was carried out as given by Guo et al. [6].

Leaf Chl and Car were determined according to Lichtenthaler [114] after extraction with 80% (*v*/*v*) acetone.

### 4.5. Measurements of HPR, DPPH Scavenging Activity, and Metabolite Concentrations

Leaf and root HPR were assayed spectrophotometrically using horseradish peroxidase (EC 1.11.1.7) and guaiacol [5].

Leaf and root DPPH scavenging activity was determined according to Sarker and Oba [115] after extraction with 80% (*v*/*v*) acetone.

Measurements of MDA were made with the improved thiobarbituric acid-reactive-substances assay after extraction with 80% (*v*/*v*) ethanol [116].

For the measurements of NCs, frozen root apices and leaves were extracted with 80% (*v*/*v*) ethanol. After centrifugation, the Suc, Glu, and Fru in the supernatant were assayed with an enzymic method [117], and the starch in the residue was measured with the anthrone reagent [118].

Measurements of TPs were made with the Folin–Ciocalteau reagent using gallic acid as a standard after extraction with 10% (*v*/*v*) methanol [119].

Total flavonoids and total COUs were measured spectrophotometrically using rutin and COU as a standard, respectively [120].

Measurements of TSPs were performed according to Bradford [121]. Leaf and root TFAAs were estimated with a ninhydrin colorimetric assay after extraction with 10% (*v*/*v*) acetic acid [119].

### 4.6. Measurements of Antioxidant Enzyme Activities

Approximately 30 mg of frozen sample was ground with a precooled mortar and pestle in 2 mL of extraction solution containing 50 mM KH_2_PO_4_-KOH (pH 7.5), 0.5% Triton X-100, 1 mM EDTA-Na_2_, and 4% insoluble polyvinylpolypyrrolidone (PVPP). After the extract was centrifuged at 4 °C and 13,000× *g* for 10 min, the supernatant was used to measure the activities of antioxidant enzymes [6]. The activities of APX, MDHAR, GuPX, and CAT were assayed as given by Rao et al. [122]. The activity of SOD was assayed according to Giannopolitis and Ries [123].

### 4.7. Statistical Analysis

The statistical significance of differences among means was tested by a two-way ANOVA (two Al levels × two COU levels) followed by LSD at *p* ≤ 0.05 using DPS 7.05 (Hangzhou Ruifeng Information Technology Co., Ltd., Hangzhou, China). Principal coordinate analysis (PCoA) was carried out with the ChiPlot (https://www.chiplot.online/, accessed on 3 March 2026). Pearson’s correlation coefficients (PCCs) were calculated with the SPSS statistical software (version 17.0, IBM Corp., Armonk, NY, USA).

## 5. Conclusions

The current research clearly demonstrated that COU50 attenuated Al^3+^ toxicity, as evidenced by improved seedling growth, Chl level, and A_CO2_, as well as less impairment of PETC at Al1.2. Further analysis suggested that reduced tissue Al concentration and enhanced capability to maintain nutrient (concentrations, distributions, and balance) and redox homeostasis functioned in COU-mediated amelioration of seedling growth inhibition, leaf Chl and A_CO2_ decline, and PETC impairment caused by Al^3+^ toxicity. Notably, COU0-treated plants exhibited some adaptive responses to Al1.2. For example, Al1.2 decreased the biosynthesis and accumulation of proteins and AAs to meet the increased need for energy; increased the DPPH scavenging activity and phenolic compound accumulation to meet the elevated demand for ROS and Al detoxification; and increased the accumulation of soluble sugars to meet the augmented demand for ROS scavenging and energy. To conclude, the research revealed some mechanisms for the COU-mediated mitigation of plant Al^3+^ toxicity. These findings lay the foundation for developing novel measurements to correct Al^3+^ toxicity in *Citrus* and other crops and for further investigating the molecular mechanisms by which COU alleviates Al^3+^ toxicity in crops.

## Data Availability

The original contributions presented in this study are included in the article/Supplementary Material. Further inquiries can be directed to the corresponding authors.

## Supplementary Materials

The following supporting information can be downloaded at: https://www.mdpi.com/article/10.3390/plants15111694/s1, Figure S1: Impacts of Al–COU treatments on the mean (±SE, *n* = 4) nutrient uptake per plant (UPP, (A–K)) and uptake per root DW (UPR, (L–V)); Figure S2: Mean (±SE, *n* = 4) ratios of N, K, Ca, Mg, and S concentrations to P concentration in roots (A–E) and leaves (F–J), as well as ratios of N, K, Ca, Mg, and S UPP to P UPP (K–O) in ‘Sour pummelo’ seedlings in response to Al–COU treatments; Figure S3: Mean (±SE, *n* = 4) distributions of nutrients in leaves (A–K), stems (L–V), and roots (W–AG) in response to Al–COU treatments; Figure S4. Impacts of Al–COU treatments on the mean (±SE, *n* = 4) HPR (A), concentrations of MDA (B), activities of APX (C), CAT (D), MDHAR (E), SOD (F), and GuPX (G), concentrations of total COUs (H), total flavonoids (I), and TPs (J), and DPPH scavenging activity (K) in leaves (above column) and roots (below column); Figure S5: Regression analysis between some parameters; Figure S6: Regression analysis between some parameters; Figure S7: Regression analysis between some parameters; Table S1: Pearson correlation coefficient matrix for the mean values of 182 physiological parameters in four Al–COU treatments; Table S2: Summary of parameters, formulae and their description using data extracted from chlorophyll (Chl) *a* (OJIP) transient.
