# Supplementary material for: Coumarin Reduces Aluminum-Induced Inhibition of Growth and Photosynthesis in Citrus grandis by Reducing Tissue Al Concentration and Maintaining Nutrient and Redox Homeostasis
Source: Plants (Basel). 2026 May 30;15(11):1694. doi: 10.3390/plants15111694 (PMC13258906; doi:10.3390/plants15111694)
Supplement: Supplementary file 1 [file plants-15-01694-s001.zip › 2026YangPlantsSupplementary Figures S1-S7.pdf]

Supplementary Figures S1-S7

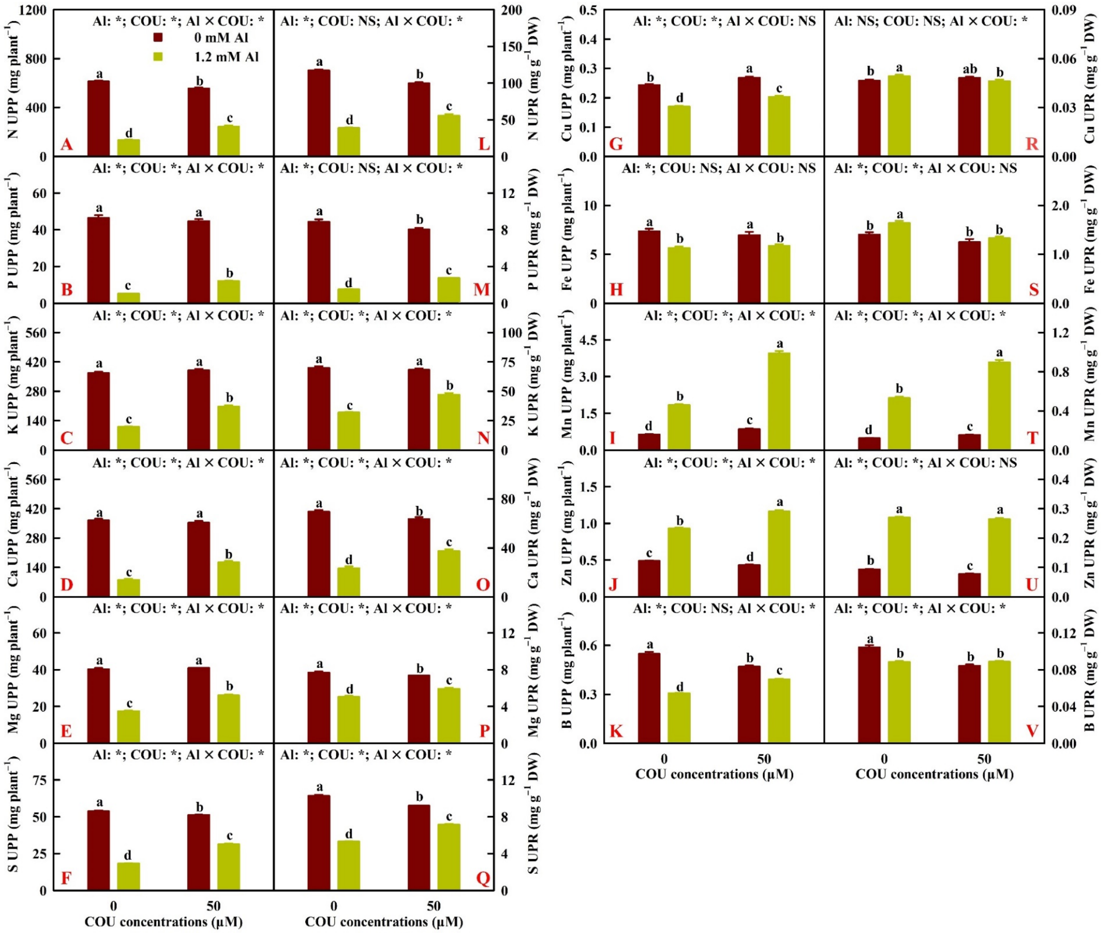

Figure S1. Impacts of Al-COU treatments on the mean (± SE, n = 4) nutrient uptake per plant (UPP, (**A-K**)) and uptake per root DW (UPR, (**L-V**)). Bars with different letters are significantly different at $p \leq 0.05$. Al: *, COU: *, and Al × COU: * represent that the F values for Al, COU, and Al × COU are significant at $p \leq 0.05$. COU: NS and Al × COU: NS represent that the F values for COU and Al × COU are not significant ($p > 0.05$).

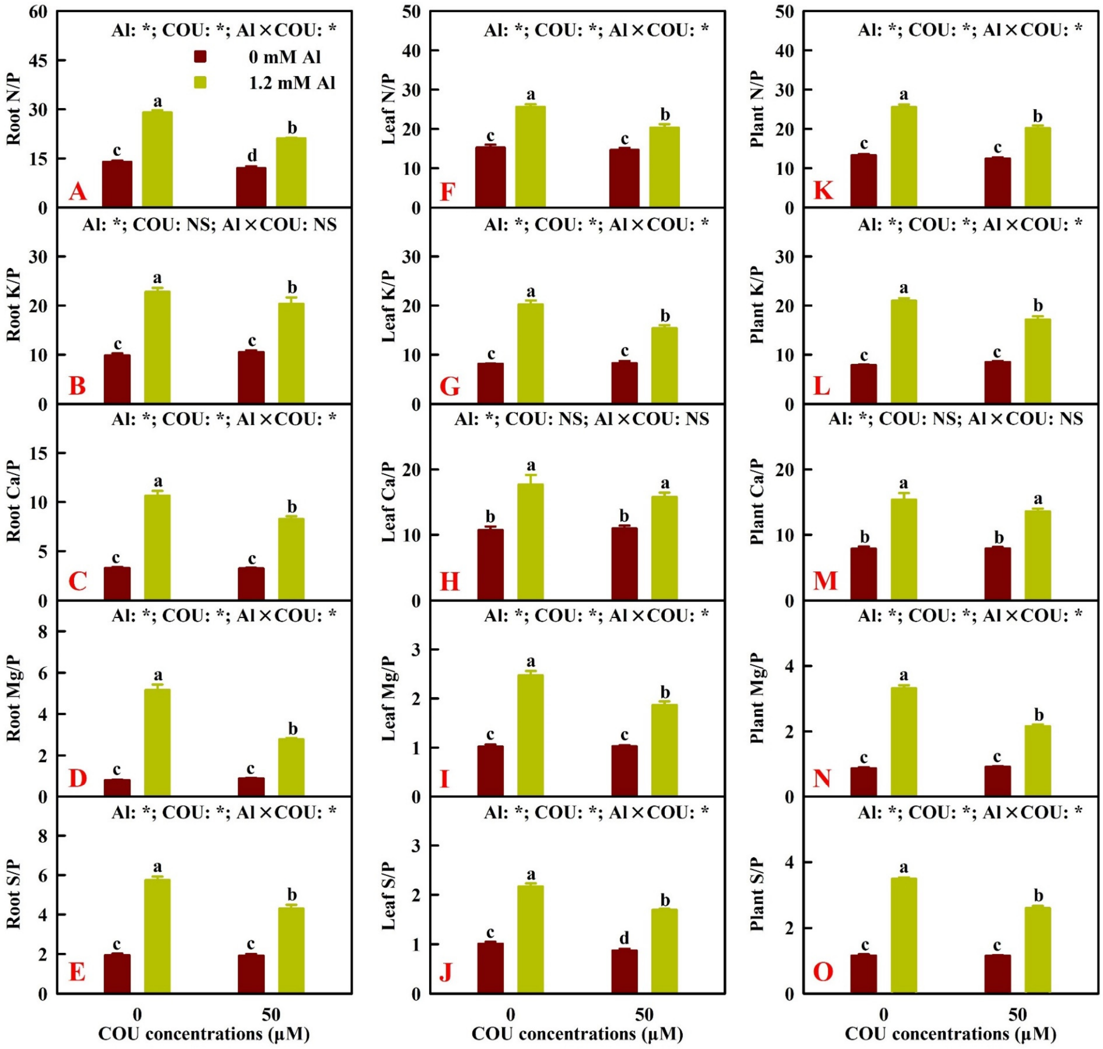

Figure S2. Mean (± SE, n = 4) ratios of N, K, Ca, Mg, and S concentrations to P concentration in roots (**A-E**) and leaves (**F-J**), as well as ratios of N, K, Ca, Mg, and S UPP to P UPP (**K-O**) in 'Sour pummelo' seedlings in response to Al-COU treatments. Bars with different letters are significantly different at $p \leq 0.05$. Al: *, COU: *, and Al × COU: * represent that the F values for Al, COU, and Al × COU are significant at $p \leq 0.05$. COU: NS and Al × COU: NS represent that the F values for COU and Al × COU are not significant ($p > 0.05$).

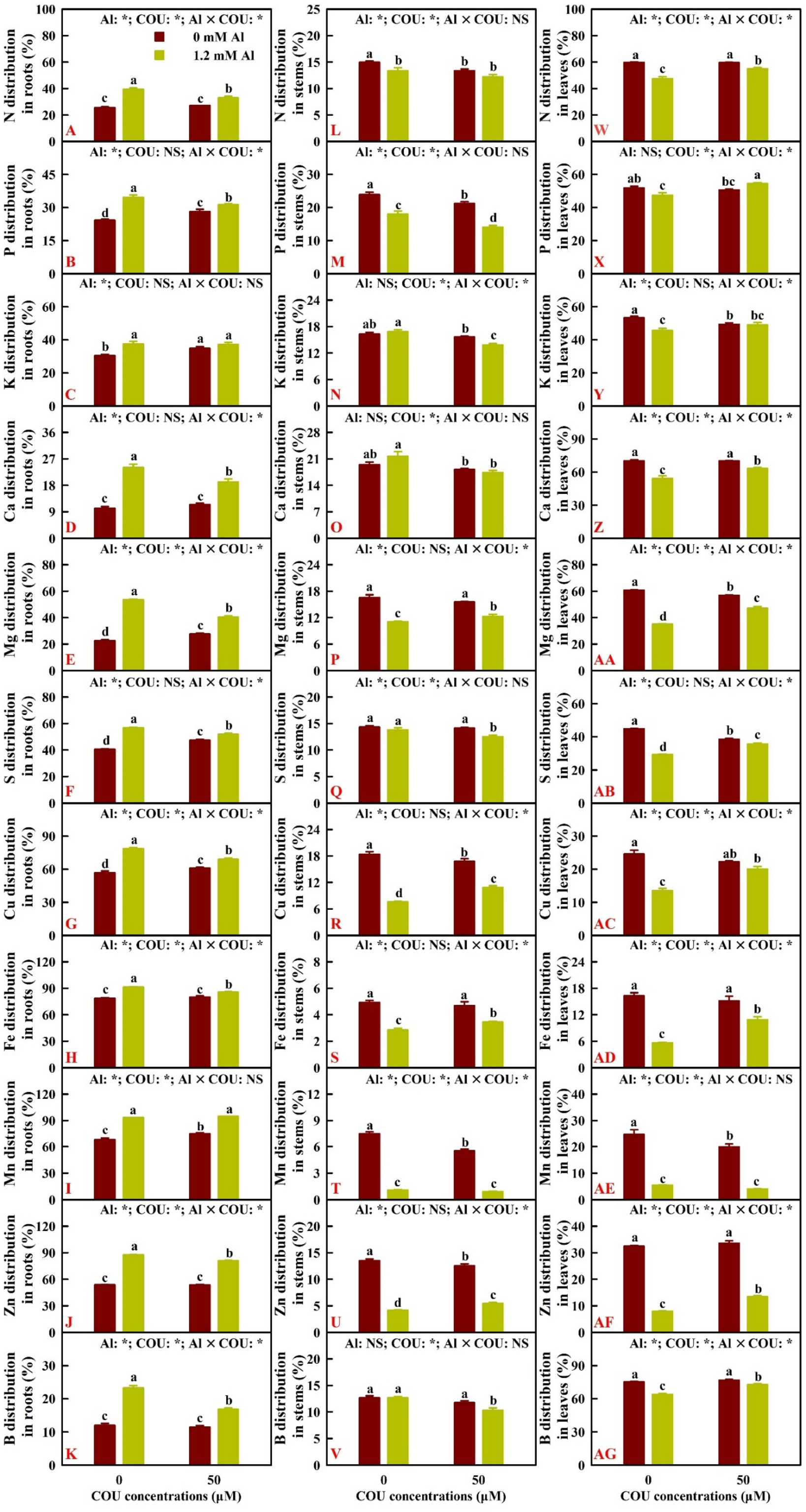

Figure S3. Mean (± SE, $n = 4$) distributions of nutrients in leaves (**A-K**), stems (**L-V**), and roots (**W-AG**) in response to Al-COU treatments. Bars with different letters are significantly different at $p \leq 0.05$. Al: *, COU: *, and Al × COU: * represent that the F values for Al, COU, and Al × COU are significant at $p \leq 0.05$. Al: NS, COU: NS, and Al × COU: NS represent that the F values for Al, COU, and Al × COU are not significant ($p > 0.05$).

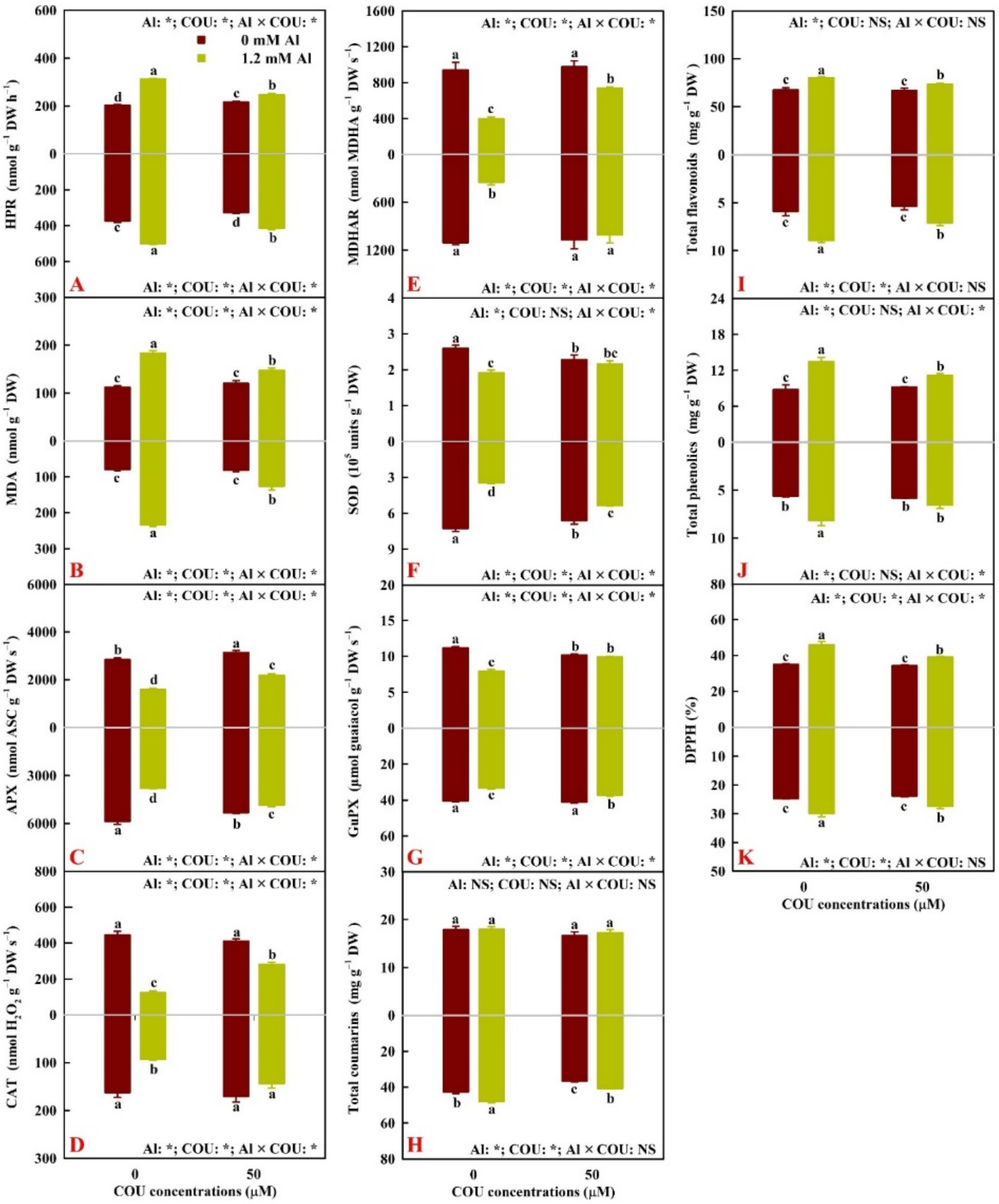

Figure S4. Impacts of Al-COU treatments on the mean (± SE, n = 4) HPR (**A**), concentrations of MDA (**B**), activities of APX (**C**), CAT (D), MDHAR (**E**), SOD (**F**), and GuPX (**G**), concentrations of total COUs (**H**), total flavonoids (**I**), and TPs (**J**), and DPPH scavenging activity (**K**) in leaves (above column) and roots (below column). Bars with different letters are statistically significant ($p \leq 0.05$). Al: *, COU: *, and Al × COU: * represent that the F values for Al, COU, and Al × COU are significant at $p \leq 0.05$. HPR, H_2O_2 production rate; MDA, malondialdehyde; APX, ascorbate peroxidase; CAT, catalase; MDHAR, monodehydroascorbate reductase; SOD, superoxide dismutase; GuPX, guaiacol peroxidase.

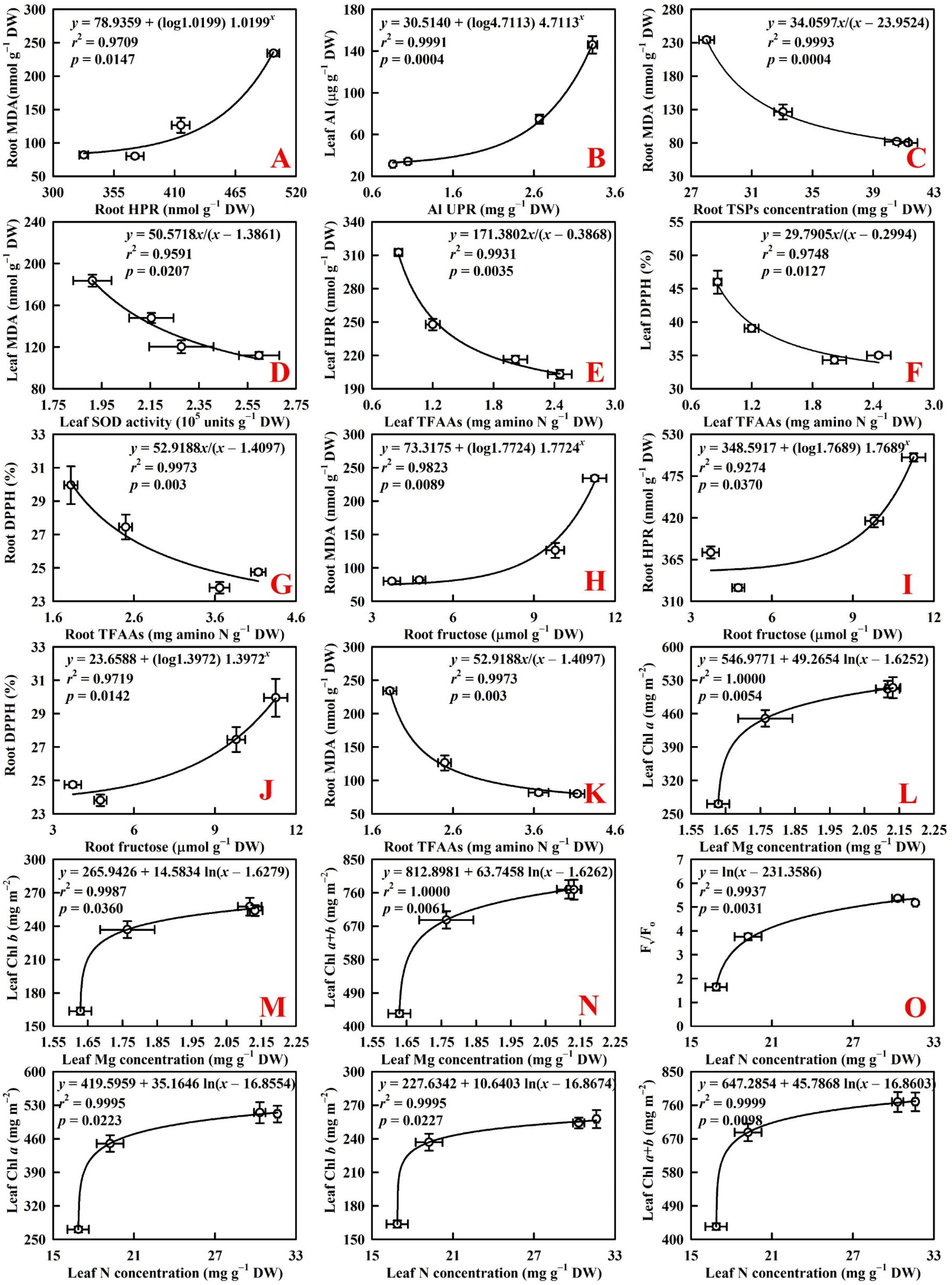

Figure S5. Regression analysis between some parameters. Each was the mean ± SE (n = 10 for fluorescence parameters or 4 for other parameters) for the independent variables (horizontal) and the dependent variables (vertical).

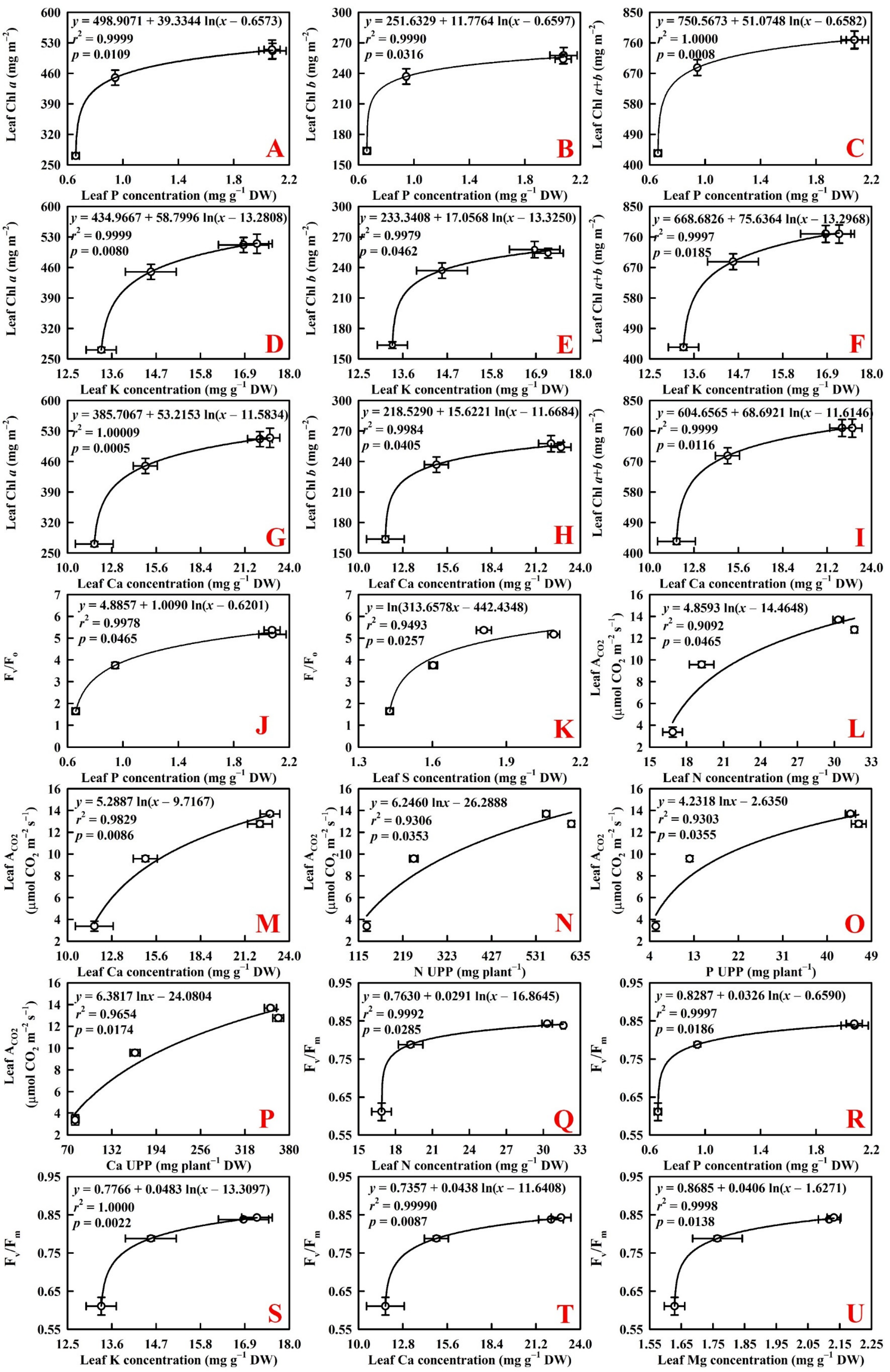

Figure S6. Regression analysis between some parameters. Each was the mean ± SE (n = 10 for fluorescence parameters or 4 for other parameters) for the independent variables (horizontal) and the dependent variables (vertical).

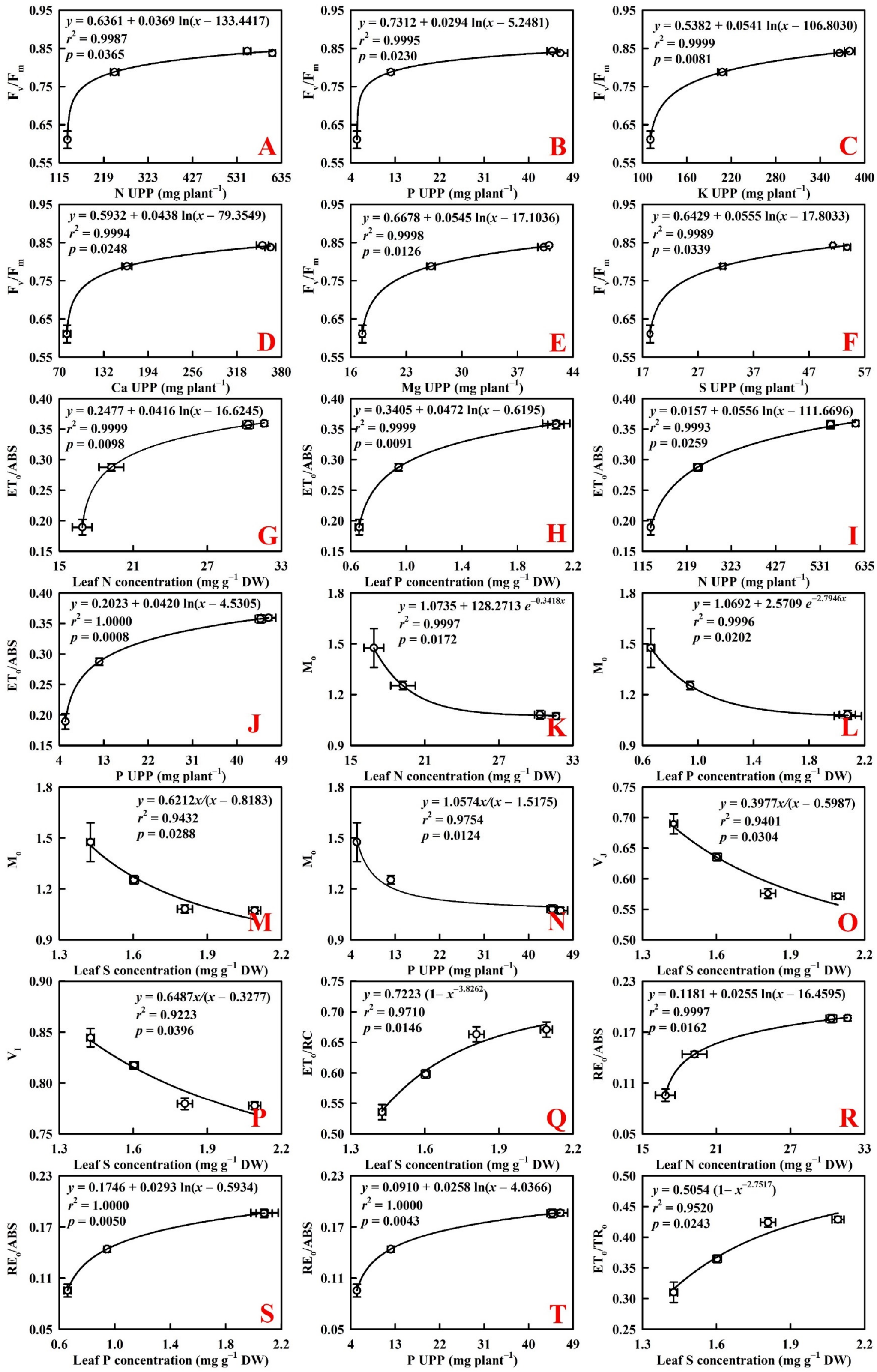

Figure S7. Regression analysis between some parameters. Each was the mean ± SE (n = 10 for fluorescence parameters or 4 for other parameters) for the independent variables (horizontal) and the dependent variables (vertical).
